# Strain-induced suppression of thermochromism in divalent cobalt molybdate thin films

**DOI:** 10.1039/d6sc04298h

**Published:** 2026-10-16

**Authors:** Kiri Van Koughnet, Joey Williamson, Kane H. J. Hill, Robert G. Buckley, Yurii G. Pashkevich, Sergej M. Orel, Eric Lebraud, Manuel Gaudon, Peter P. Murmu, Sarah Spenser, Dominique Appadoo, Shen V. Chong, Fryderyk Lyzwa

**Affiliations:** a Robinson Research Institute, Victoria University of Wellington P. O. Box 33-436 Lower Hutt 5046 New Zealand bob.buckley@vuw.ac.nz shen.chong@vuw.ac.nz; b Department of Physics, Photon Factory, University of Auckland 38 Princes Street Auckland 1010 New Zealand f.lyzwa@auckland.ac.nz; c Te Whai Ao Dodd-Walls Centre for Photonic and Quantum Technologies New Zealand; d MacDiarmid Institute for Advanced Materials and Nanotechnology P. O. Box 600 Wellington 6140 New Zealand; e Department of Physics and Fribourg Center for Nanomaterials, University of Fribourg Chemin du Musée 3 Fribourg Switzerland; f O. O. Galkin Donetsk Institute for Physics and Engineering NAS of Ukraine 03028 Kyiv Ukraine; g Univ. Bordeaux, CNRS, Bordeaux INP, ICMCB, UMR5026 87 Avenue du Dr A. Schweitzer Pessac F-33608 France; h National Isotope Centre, Earth Sciences New Zealand 30 Gracefield Rd, PO Box 30368 Lower Hutt 5010 New Zealand; i THz/Far-IR Beamline, Australian Synchrotron 800 Blackburn Rd Clayton VIC 3168 Australia

## Abstract

Thermochromic oxides provide a platform for coupling lattice, electronic, and magnetic degrees of freedom, with divalent cobalt molybdate (CoMoO_4_) as a prototypical example. Despite extensive studies on powders, its thin-film behaviour – critical for device applications – remains largely unexplored. Here, we present a combined experimental and theoretical investigation of CoMoO_4_ thin films, including the first terahertz-to-visible/UV optical spectra of β-CoMoO_4_ in thin-film form. In contrast to bulk powders, which undergo a first-order β → α structural transition near 230 K, the thin films retain the high-temperature β-phase across the entire temperature range. We observe that our thin films exhibit an altered magnetic behaviour compared to that of classical powder samples with the same chemical composition. We also show that microstrain fundamentally reshapes the phase landscape, suppressing thermochromism and stabilizing the high-temperature phase. The optical spectra reveal pronounced anomalies in the lattice and electronic responses, including a softening of a low-energy, cation-dominated phonon (42 cm^−1^) upon cooling, in contrast to conventional hardening of higher-energy modes. This behaviour indicates incipient atomic displacements analogous to those driving the bulk β → α transition, despite the absence of a structural phase change, and saturates between 200 and 150 K. Temperature-dependent X-ray diffraction confirms persistent β-phase symmetry with increasing microstrain, consistent with strain-induced frustration of the first-order transition. At higher energies (0.12–3.7 eV), the optical response exhibits a blue-shift of Co^2+^ crystal-field transitions and charge-transfer excitations, indicating a strain-enhanced ligand field. Supported by structural refinements and theoretical calculations, we identify crystal field strengthening as the key mechanism stabilising the β-phase. These results establish strain as a thermodynamic lever to control phase stability and functional properties in thermochromic oxides.

## Abstract 2

Thermochromic oxides provide a platform for coupling lattice, electronic, and magnetic degrees of freedom, with divalent cobalt molybdate (CoMoO_4_) as a prototypical example.

## Graphical abstract

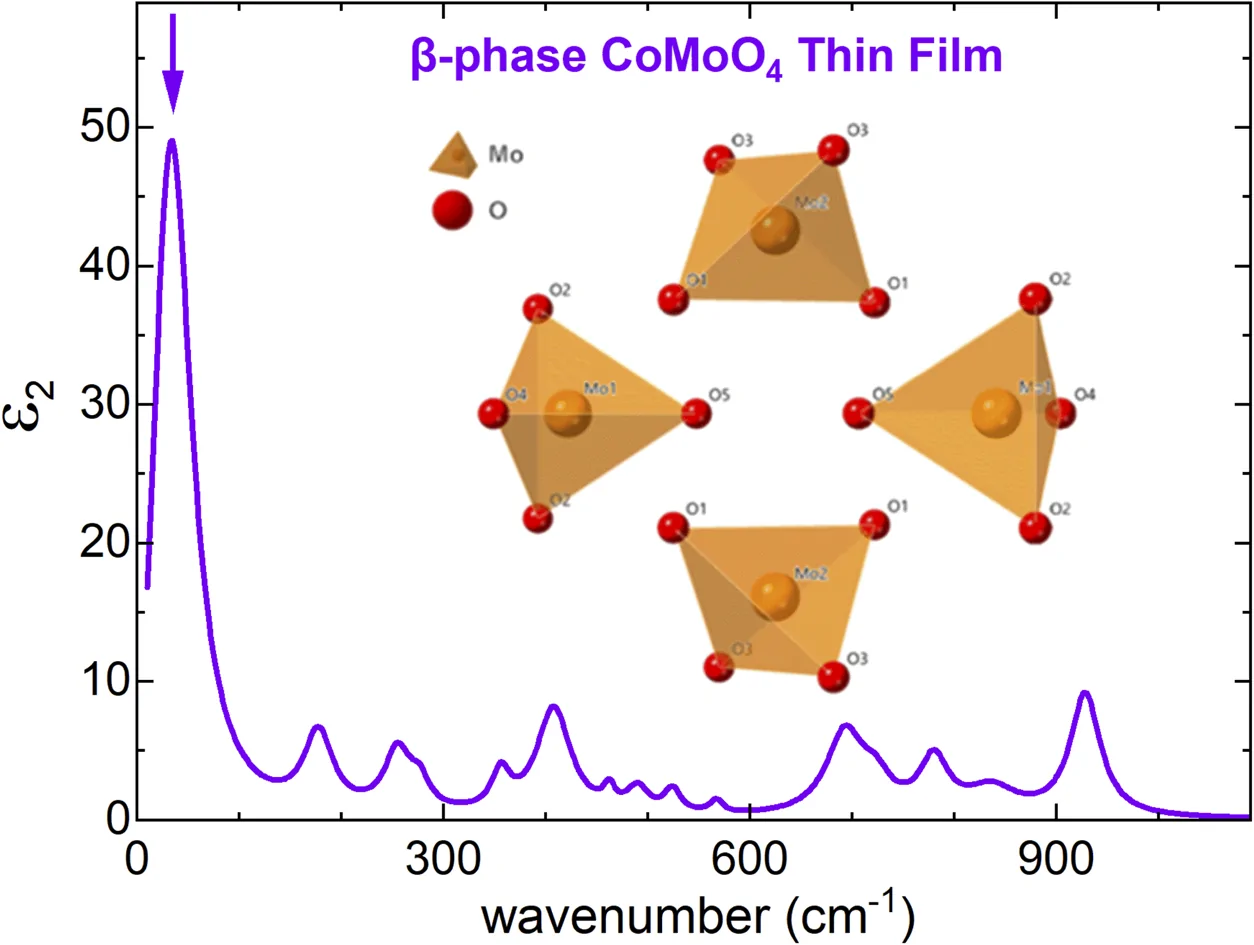

## Introduction

Allotropic phases of the divalent molybdates display contrastingly changing colours while exhibiting different magnetic states.^1–4^ This range of interesting behaviours has been exploited in numerous applications, including catalysis, batteries, and sensing^5–7^. Of particular interest is the prominent colour change exhibited by the high-pressure and low-temperature divalent molybdate α-CoMoO_4_ phase, changing from green to the structurally different purple β-CoMoO_4_ high-temperature and low-pressure phase; both phases of CoMoO_4_ are stable under ambient conditions.^1,3^ For powder CoMoO_4_, the transition temperatures between these two phases have been measured through optical, XRD and Raman measurements, revealing a hysteretic phase transition: *T*(β → α) at 230 K upon cooling and *T*(α → β) at 780 K upon heating^1–3^. Both phases crystallise in the monoclinic *C*2/*m* space group and have Co^2+^ ions in a distorted octahedral site (CoO_6_).^1–3,8^ The two phases differ in the coordination of their Mo^6+^ ions: β-CoMoO_4_ has a structure in which the Mo^6+^ cations form isolated distorted tetrahedra (MoO_4_), whereas α-CoMoO_4_ has a structure where the Mo^6+^ ions form Mo_4_O_16_ tetramers *via* edge sharing, leaving the Mo^6+^ ion in a distorted octahedral site (MoO_6_).^1–3,8^Fig. 1 displays the edge sharing Mo_4_O_16_ tetramers and isolated MoO_4_ tetrahedra for α-CoMoO_4_ and β-CoMoO_4_, respectively.

**Fig. 1:**
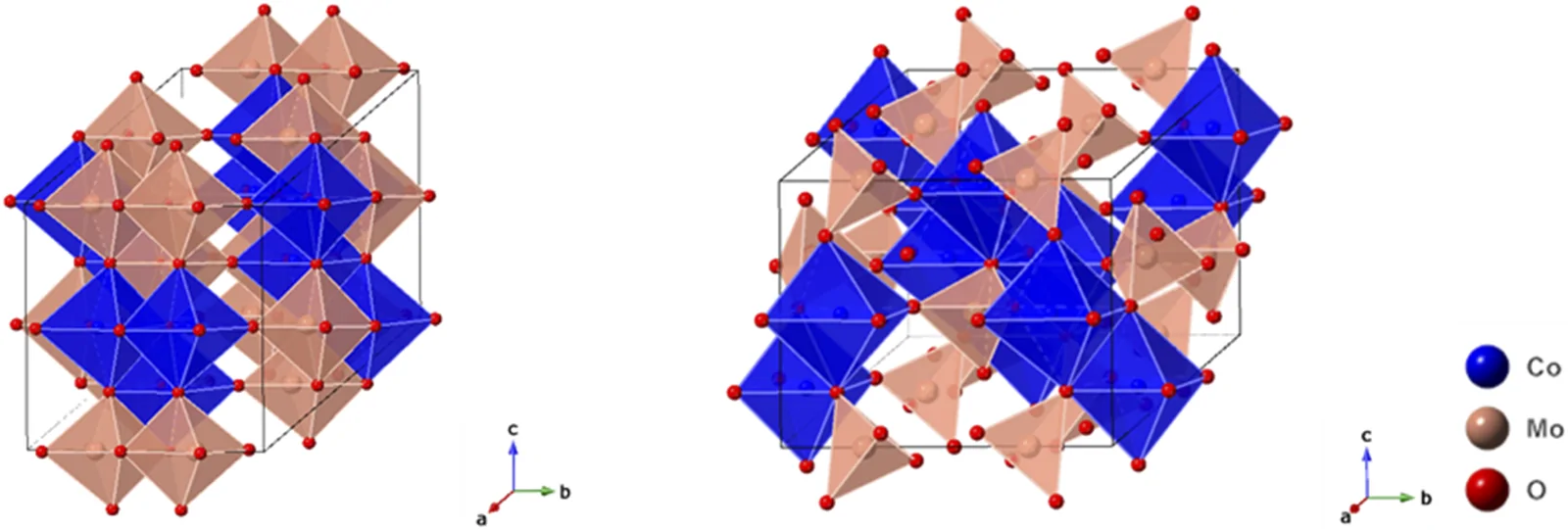
Crystal structure of CoMoO_4_. Images are generated by CrystalMaker, illustrating Mo_4_O_16_ tetramers in the α-phase consisting of four edge-shared MoO_6_ octahedra (left) and the isolated MoO_4_ tetrahedra in the β-phase (right).

Most of the work reported on CoMoO_4_ was undertaken on powder samples.^1,3,8,9^ Studying single crystals, instead of powders, would be ideal for investigating the electronic and magnetic properties of CoMoO_4_ arising from anisotropy. However, the potential for cracking and fracturing during temperature-dependent studies undergoing the thermochromic first-order phase transition of this molybdate between α and β phases may introduce signal artefacts. This is because, upon cooling (β → α) or heating (α → β), CoMoO_4_ undergoes a 6% change in the unit cell volume^3^, which as observed in single crystal CuMoO_4_ (ref. 10) and CuMoO_4_ crystallites,^11^ may lead to cracking of the crystals. Another issue is that large millimetre-sized CoMoO_4_ single crystals are not readily available.^12–14^ For many applications, however, thin films would be the material form of choice. However, there have only been a few studies based on CoMoO_4_ thin films in the literature,^15–17^ including our recent report on the magnetic properties of mixed α- and β-phase CoMoO_4_ thin films.^18^

Here, we report a combined structural, magnetic and optical study of thin films of one phase, namely β-CoMoO_4_, deposited on silicon and sapphire substrates. Interestingly, we find that the β-phase is stable in thin film form down to the lowest temperatures (*T* = 12 K), as confirmed by X-ray diffraction (XRD). To enable the utilisation of β-CoMoO_4_ for applications, we explored the low-energy excitations (infrared-active phonons) and electronic transitions in pure β-CoMoO_4_ thin films and compared the findings with the properties of powder samples. To this end, and because of the enhanced stability of thin films of the β-phase, we report the first optical spectra of β-CoMoO_4_ in thin film form across a wide spectral range from the terahertz to the visible/UV. We studied the temperature dependence of the phonons between 300 K and 150 K and observed an anomalous softening of the lowest energy (42 cm^−1^) cation-dominated phonon. In the visible/UV spectral range we observe the 300 K behaviour across spectral regions, probing the Co^2+^ d–d band transitions and the oxygen 2p- to- Mo-d-orbital inter-band or charge transfer transition.

## Experimental methods

### Film deposition

β-CoMoO_4_ films were deposited at room temperature using magnetron sputtering on a variety of substrates. XRD and magnetic studies were carried out on films deposited on silicon substrates with a 200–300 nm thermally grown SiO_*x*_ surface layer. For the optical studies, films were deposited on double-side polished silicon and sapphire substrates. The 1-inch CoMoO_4_ sputtering target, with a purity of 99.9%, was supplied by ACI Alloys, Inc. (USA). In all cases, the films were grown using RF magnetron sputtering with a power of 30 watts at room temperature, under a partial pressure of 6 mTorr of argon, and post-deposition annealed *in situ* at 700 °C for 5 hours in 1 mTorr of oxygen. For a target film thickness of 200 nm, the film was deposited for 1 hour. To improve our sensitivity for the optical study, we increased the deposition to, for example, 2-hour deposition duration to achieve a film thickness of 450 nm. The actual film thickness was determined by scanning electron microscopy employing a Hitachi SU7000 system, where it was found that a target film thickness of 200 nm resulted in a measured thickness of 185 ± 15 nm and that of the thicker film in a thickness of 470 ± 20 nm. Thin films with various thicknesses were grown to improve the sensitivity of the different experimental probes employed. Herein, we will refer to the films by their nominal thicknesses of 200 nm, 290 nm and 450 nm, unless otherwise specified.

### Structural and compositional characterisation

X-ray diffractograms were acquired on a Rigaku Smartlab system using Co K_α_ X-ray radiation at a wavelength of 1.7889 Å at a grazing angle of incidence (2°) to suppress any contribution from the substrate. Temperature-dependent XRD patterns were collected between room temperature and 12 K on a PANalytical X'Pert Pro powder diffractometer equipped with an Oxford Instruments liquid helium cryostat (6K) employing copper radiation in the usual *θ*–2*θ* configuration.

The compositional profile of the β-CoMoO_4_ film was determined using Rutherford backscattering spectrometry (RBS). RBS was performed with a 2.0 MeV ^4^He^+^ probe beam, a beam diameter of 1 mm, and a surface barrier detector positioned at a 165° backscattering angle to measure the energy and yield of backscattered particles. With this probe energy, the depth profile will be in the µm range; therefore, RBS provides the average composition of the entire 200 nm thick cobalt molybdate film. The film's composition was determined using the Rutherford Universal Manipulation Program (RUMP) computer simulations.^19^

X-ray photoelectron spectroscopy (XPS) measurements were conducted on an ESCALAB250Xi Thermo Scientific XPS system using monochromatic Al Kα radiation (1486.6 eV) set at 120 W. The measurements were undertaken with perpendicular incident X-rays and a photoelectron take-off angle of 90°, enabling a depth profile of ≤50 Å. XPS scans were acquired with a step size of 0.1 eV and with a pass energy of 20 eV. The binding energy was referenced to the XPS C 1s peak of adventitious carbon at 284.4 eV. The quantification of atomic compositions was obtained from the XPS peak areas using a Shirley background function with built-in cross-sections adjusted.

### Magnetic characterisation

Magnetisation data on the CoMoO_4_ thin films were acquired using a Quantum Design MPMS-7 SQUID magnetometer with the applied magnetic field parallel to the surface of the films and performing measurements between 2.2 K and 300 K in magnetic fields up to 6 tesla. Note that the magnetisation curves presented have not been background subtracted.

### Optical measurements

We conducted comprehensive optical characterisation of the CoMoO_4_ films, ranging from terahertz (THz) to the ultraviolet (UV) spectral range. Across this wide spectral range, we employed two substrates: silicon in the THz-mid-infrared and sapphire in the near-infrared/visible/UV.

The THz/far-infrared transmission experiments were performed at the Australian Synchrotron's Terahertz beamline. Transmission spectra were measured between 20 and 685 cm^−1^ (2.5–85 meV) at a resolution of 1 cm^−1^ using a Bruker IFS125 spectrometer equipped with a 6 µm Mylar beamsplitter and a Si bolometer. The samples were mounted on the translation stage of a cryostat optically coupled to the IFS125 spectrometer and studied from room temperature down to 150 K. These spectra were combined with 300 K infrared (IR) to ultraviolet (UV) spectra, ranging from 150 cm^−1^ (18 meV) to 30 000 cm^−1^ (3.7 eV) and acquired at a laboratory-based Bruker Vertex 80v interferometer, measuring both the reflectivity and transmission. The technique has been reported elsewhere.^20,21^ Further information on the signal intensity can be found in the SI in Fig. S1. The spectral transmission was determined by calculating the ratio of the transmitted and incident signal intensities, while the reflectivity was determined relative to an evaporated Al film and corrected by employing literature values for the aluminium reflectivity.^22^ The reflectivity was measured at near-normal incidence to the sample surface plane to study the film's in-plane response. The uncertainty in the magnitude of these quantities is ±2%, based on the reproducibility of the spectra.

The reflectivity and transmission spectra of the film-substrate stack were simultaneously modelled, by means of the public domain software package RefFit.^23^ By using a Kramers–Kronig consistent sum of Drude–Lorentzian functions, we simulate the energy-dependent dielectric function, *ε*(*ω*) = *ε*_1_(*ω*) + i*ε*_2_(*ω*), where:1
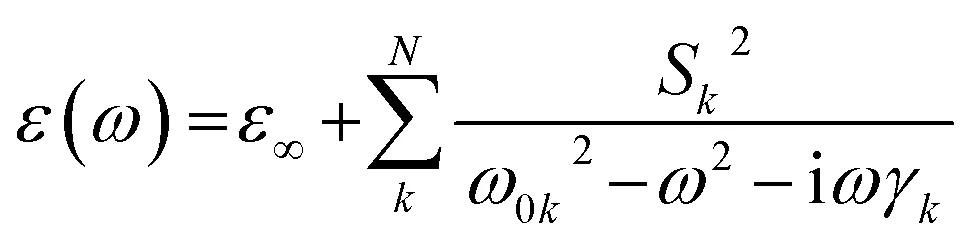
Here, *ε*_∞_ represents the high-frequency dielectric constant, *ω* is the wavenumber, *ω*_0_ is the oscillator frequency, *S* is the oscillator strength, *γ* is the damping coefficient for each oscillator *k*. The optical response of the substrate was incorporated after performing independent measurements of *R* and *T* of a bare substrate, *i.e.* of either silicon or sapphire, and then deriving a model substrate dielectric function. The film thickness, as determined above, was used as a fixed parameter.

## Results and discussion

### Sample growth, structure, and chemical composition

Fig. 2(a) exhibits an XRD pattern collected at a 2° X-ray incident angle at ambient temperature for a 200 nm film grown on a Si substrate. The XRD pattern confirms the exclusive presence of β-phase CoMoO_4_, showing all the reflections that are expected for a monoclinic crystal structure with a space group of *C*2/*m*.^24^ Notably, there are no additional XRD reflections from impurities or Bragg peaks at 16.4° and 33.1° 2*θ* for (110) and (220) Miller indices, respectively, which would indicate the presence of α-CoMoO_4_. Furthermore, like the powder XRD patterns found in previous studies,^1,2^ there is a preferential growth along the (002) plane at a 2*θ* of 30.7°, as indicated in Fig. 2(a). The 300 K lattice parameters of a monoclinic β-phase CoMoO_4_ film on silicon are listed in Fig. 2(b) and are in good agreement with the values found previously for films on both silicon and sapphire and for powder samples.^18^ A few films did, however, exhibit a minor (4%) fraction of α-CoMoO_4_, which we will discuss further below.

**Fig. 2:**
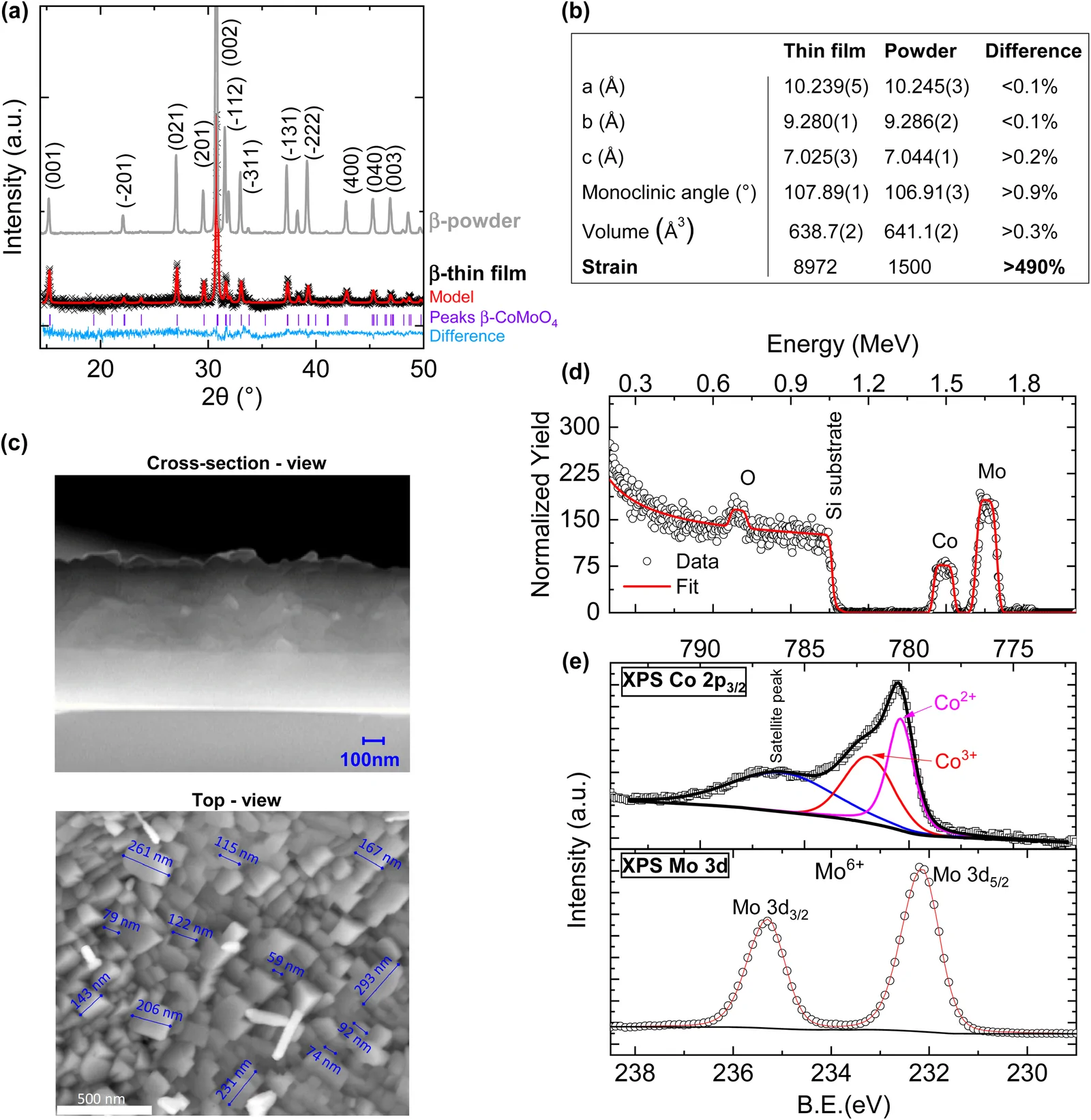
Growth, structure and chemical composition of β-phase CoMoO_4_ thin films. (a) Room temperature (300 K) experimental XRD pattern for the 200 nm-thick film grown on Si, and β-phase CoMoO_4_ powder plotted for comparison. The red-coloured trace shows the calculated XRD pattern for the film obtained from structural refinement using GSAS; the resulting lattice parameters (film and powder) are listed in (b). (c) SEM cross-sectional view and top view of a 450 nm-thick β-CoMoO_4_ film showing the formation of densely packed sub-micron crystallites. A thicker film was employed for ease of imaging. (d) Rutherford backscattering spectrum, (e) XPS Co 2p_3/2_ and Mo 3d core level of a 200 nm-thick β-CoMoO_4_ thin films for identification of the chemical composition and oxidation states.

In addition, temperature-dependent XRD patterns were collected between 300 K and 12 K for two different thin films deposited on silicon substrates (Fig. S2 and S3). These demonstrate that, unlike for powder samples, the β-phase CoMoO_4_ thin film is stable down to 12 K and that the samples do not exhibit a thermochromic phase transition at ∼230 K. The reflections attributed to the small fraction of α-CoMoO_4_ observed in one of the films (∼4%, as noted above and detailed in Fig. S3) also remain unchanged in both position and intensity down to 12 K. While we observed the expected contraction of the β-phase unit cell volume with decreasing temperature, the contraction is not uniform, saturating at 150 K with no measurable change between 150 K and 12 K (Table S1). The measured changes in the *a*- and *c*-axes were not significant, but there was an observable contraction of 0.1% in the *b-*axis between 300 K and 150 K and a 0.9% decrease in the monoclinic angle. The same temperature independence was seen for the minor α-CoMoO_4_ phase.

To elucidate the absence of thermochromism in our films, we undertook an SEM study of the film morphology. Fig. 2(c) shows a typical SEM micrograph illustrating the formation of densely packed sub-micron crystallites in our films that range from 60 to 320 nm. Initial inspection of the particle size distribution in these films indicates that ∼60% of the grains have linear dimensions above 100 nm, so the volume of the material at that larger dimension dominates the size distribution. While bulk behaviour dominates for crystallite sizes of ≥100 nm,^25^ the surface energy effects associated with a reduced grain size only start to become significant for crystallite sizes notably below 100 nm. The SEM results thus indicate that surface-energy effects associated with a reduced grain size are not playing a significant role in stabilising the β-CoMoO_4_ phase in these films. Studies on CoMoO_4_ (ref. 5 and 26) and CuMoO_4_ (ref. 27) have further shown that low-dimensional structures tend to stabilise low-coordination structures, namely the tetrahedral MoO_4_ in CoMoO_4_ and CuMoO_4_ and the square-pyramidal CuO_5_ in the high-temperature CuMoO_4_ phase. As the particle size decreases, the ability of these low-coordination structures to transform into the respective octahedral coordination (MO_6_) at low temperatures becomes increasingly challenging.

The dominant role of strain in suppressing the phase transition in our CoMoO_4_ films is illustrated by the temperature-dependent XRD patterns (Table S1) that were obtained from structural refinements (GSAS-II^28^). This allowed us to estimate a value for the microstrain.^29^ The instrumental broadening was calculated from measurements of a NIST SRM1976 Al_2_O_3_ standard reference. The particle size parameter was fixed at 4 µm for all refinements such that non-instrumental broadening effects were modelled solely by the microstrain parameter. While the resulting absolute values for the microstrain are not representative of the actual strain occurring in the sample, the temperature-dependent changes well-reflect the actual changes in the strain. Following this approach, the estimated microstrain value increased by 3.7% as the samples were cooled to 150 K and saturated at lower temperature, resulting in a total 4.5% strain-change between 300 K and 12 K. This demonstrates that microstrain in the films increases with decreasing temperature across the expected thermochromic transition temperature, despite the absence of the transition in these thin films. Analysis of 300 K XRD measurements on β-phase CoMoO_4_ powder, exhibiting a thermochromic phase transition at ∼230 K, also shows that microstrain is 4–5 times larger in thin films than in powders of β-CoMoO_4_, providing further evidence for an enhanced strain state in the films. While the factors that govern the irreversibility of the β → α transition remain an open question,^1^ our results indicate that strain plays an important role, consistent with the arguments of ref. 3.

Further composition analyses of bulk and the surface properties were carried out using Rutherford backscattering spectrometry (RBS) and X-ray photoelectron spectroscopy (XPS), respectively. Fig. 2(d) shows that the best-fit RUMP-simulated RBS spectrum was obtained using a composition ratio of Co : Mo : O = 1 : 1 : 4, consistent with the film's target stoichiometry as CoMoO_4_. XPS probes the surface electronic states of the CoMoO_4_ films, and Fig. 2(e) shows the XPS Co 2p spectrum which exhibits two Co 2p_3/2_ and Co 2p_1/2_ peaks at 780.6 eV and 796.6 eV, respectively. Our Co 2p_3/2_ peak was modelled *via* three components rather than two, in agreement with ref. 12 and 30. We identified two cobalt species at binding energies of approximately 780.5 ± 0.2 eV and 782.5 ± 0.2 eV, along with a shake-up satellite at 787.1 ± 0.3 eV. The lower binding energy peak is assigned to Co^2+^, supported by an observed spin–orbit splitting (SOS) of 16.0 eV, while the higher energy peak corresponds to Co^3+^, which typically exhibits a slightly lower SOS of around 15.5 eV (ref. 31) (Fig. S4). The Co^3+^ species is low-spin (*S* = 0, diamagnetic) and thus contributes negligibly to the magnetic signal (as discussed in the following magnetisation Section Fig. 3), whereas Co^2+^ is in the high-spin state (*S* = 3/2). The ratio of Co^2+^/Co^3+^ ≈1 suggests an absence of the Co_3_O_4_ species (with Co^2+^/Co^3+^ ≈0.5 (ref. 32)) at the surface of our thin films.

**Fig. 3:**
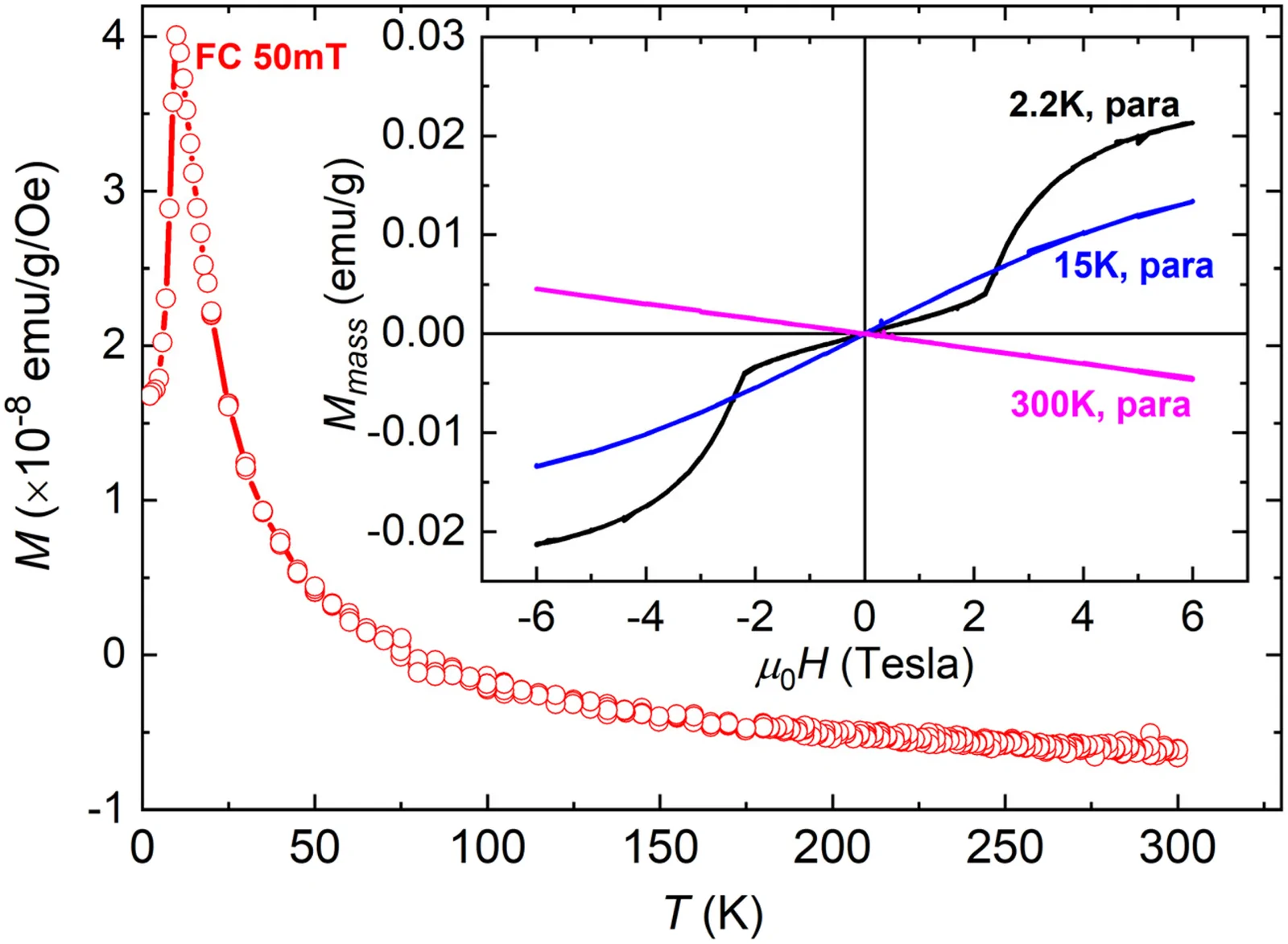
Magnetic behaviour. Temperature-dependent magnetisation of a CoMoO_4_ film exhibiting an antiferromagnetic transition (AFM) at ∼10 K. Inset: field-loops up to ±6 tesla, with the applied field being parallel (para) to the film surface and at different temperatures; hysteresis below *T*_AFM_ is absent.

The two additional peaks centred at 232.2 eV (235.3 eV) are attributed to Mo 3d_5/2_ (Mo 3d_3/2_), as shown in the bottom panel of Fig. 2(e). The spin–orbit-splitting between these two peaks is 3.1 eV, and only one species could be fitted to this peak with a FWHM ∼0.83 eV that is assigned to the fully oxidised Mo^6+^ oxidation state. No occurrence of Mo^4+^ could be observed in any of the thin films. The lattice oxygen (O^2^) concentration (not shown) relative to that of molybdenum (O/Mo atomic ratio) is 4.3 ± 0.2, which is consistent with a MoO_4_^2−^ anion. This and the results from grazing angle XRD confirm the absence of Co_2_Mo_3_O_8_ (with Mo^4+^ species), which tends to form in a low-oxygen partial pressure environment. The excess oxygen in the O/Mo ratio can also explain the presence of higher oxidised Co^3+^ species observed in the XPS Co 2p spectrum. We also note that the line shape of the shake-up satellite in the Co 2p_3/2_ trace has a characteristic flat peak with a steep edge on the high binding energy side. This distinct shape is characteristic of CoMoO_4_, as Co_3_O_4_ and CoO have different line shapes.^32,33^ Hence, the XPS results confirm the formation of a slightly oxidised stoichiometric CoMoO_4_ thin film. In view of this, we performed additional control experiments to investigate whether the presence of Co^3+^ would influence the phase transition in CoMoO_4_ thin films. We annealed the films under various conditions (argon gas flow and a dynamic vacuum of 1 × 10^−3^ torr), which would restore a Co^2+^ oxidation state. The results are shown in Fig. S5 of the SI: the grazing-incidence XRD measurements showed no evidence of α-CoMoO_4_ reflections in films annealed in argon or vacuum that were first cooled down to 2.2 K and subsequently warmed back to room temperature. This provides further evidence, together with the magnetisation data presented below, that Co^3+^ plays a negligible role in the phase transition of β-CoMoO_4_ thin films.

### Magnetism

Fig. 3 displays the field-cooled (FC) magnetisation behaviour, recorded at 50 mT. The magnetisation *M* increases with decreasing temperatures, increasing to a peak at approximately 10 K, which is due to the onset of antiferromagnetic (AFM) order.^18^ The inset shows the field loops acquired below and above the magnetic ordering temperature of 10 K. The field loops below 10 K show a single sharp magnetic transition at ±2.2 tesla without hysteresis in the low field regions (Fig. S6). The absence of hysteresis in these thin film samples highlights a difference compared to our earlier study on powder samples.^18^ Even thin films that were annealed under various conditions (as stated in the previous section) do not exhibit any hysteresis which would be characteristic of the α-CoMoO_4_ phase (data are shown in Fig. S6(c)). This behaviour is also consistent with the XRD results, supporting the absence of a transition to the α-phase down to 2.2 K.

Field-dependent magnetisation measurements on Mn- and Ni-molybdate single crystals revealed two spin–flop transitions with the applied magnetic field (*H*) being parallel to the easy-axis (*D*) and only one transition when *H*⊥*D*.^34,35^ Polycrystalline CuMoO_4_ demonstrated the existence of two spin transitions.^36^ Unlike these other molybdates, the field-dependent spin transition observed in our β-CoMoO_4_ films is sharp (even with randomly oriented crystallites) and therefore cannot be attributed to spin–flop transitions caused by weak magneto-crystalline anisotropy in MnMoO_4_ and NiMoO_4_,^34,35^ nor breaking of the AFM exchange terms in CuMoO_4_.^36^ Instead, the observed spin transition is reminiscent of a ‘spin–flip’ transition, which is mediated by a strong magneto-crystalline anisotropy and has been previously observed in other AFM materials.^37,38^ The scenario of a spin–flip (rather than a spin–flop) in our β-CoMoO_4_ films can occur if the anisotropy terms are comparable or stronger than the AFM ones. Note that, even though the magnetic structure has been studied for several molybdates in the α-phase (*via* neutron scattering or Mössbauer spectroscopy) such as for iron molybdate,^39^ it has not been explored in cobalt molybdate in the strained β-phase.

For our polycrystalline β-CoMoO_4_ thin films, we propose a large magnetic anisotropy and an Ising-like antiferromagnetic (AFM) order to account for the sharp spin–flip and the observed gradual increase in magnetisation above the transition field, *H*_c_ (at 2.2 T). This is shown in Fig. S7. One can further estimate the Zeeman energy, *E*_flip_, for decoupling AFM-ordered pairs into ferromagnetic (FM)-ordered pairs:

2 *E*_flip_ = *g*_m_*µ*_B_*H*_c_ = 7.2–7.9 cm^−1^,

using the calculated maximal *g*-factor *g*_m_ = 7–7.7 (see eqn (S3)) and the experimental value of the spin transition field, *H*_c_ = 2.2 T, at which the spin–flip starts. As expected, this energy approximately corresponds to the AFM order energy *k*_B_*T*_N_ = 7 cm^−1^ at the experimental *T*_N_ = 10 K. Indeed, an applied field of *H*_c_ = 2.2 T is sufficient to suppress the effective AFM exchange field and transfer the system into the FM state. This simple estimation provides additional evidence in favour of the Ising-like AFM structure that is realised in the CoMoO_4_ film with a strained β-phase.

### Optical spectroscopy: synchrotron-based terahertz combined with bench-top IR-UV spectra

#### Low-energy excitations: infrared-active phonons

Identifying literature studies on phonons of β-CoMoO_4_ has proved challenging, as noted in ref. 40. This is largely due to the relatively high pressures required to form large enough pellets needed for measuring infrared (IR) spectra of powder samples. These pressures are sufficient to induce a transition from the β-phase to α-CoMoO_4_. These previous IR reports are limited to room-temperature studies of α-CoMoO_4_.^40,41,42,43^ Here we present, to the best of our knowledge, the first IR study of β-CoMoO_4_, including an investigation of its temperature-dependent behaviour.

Fig. 4(a and b) show the room temperature measured terahertz to infrared transmission (*T*) and reflectivity (*R*) spectra (black curves), across the IR-active phonon region for a 450 nm thick β-CoMoO_4_ film grown on a silicon substrate. As noted above, the transmission is a combined data set collected on the Australian Synchrotron (20 cm^−1^ to 1200 cm^−1^) and a benchtop-based system (150 cm^−1^ to 1200 cm^−1^). In total we detected 16 absorption bands in the transmission spectrum, which can be assigned to phonons and will be discussed below. The results of simultaneously modelling *T* and *R* (in red) are also displayed in Fig. 4(a) and (b), respectively. As described above, this spectral region was modelled using a series of Lorentzian oscillators to describe the phonon behaviour of β-CoMoO_4_. The resulting energy-dependent real and imaginary parts of the dielectric function are plotted in Fig. 4(c) and (d). Interestingly, the derived dielectric function demonstrates that the low-energy absorption band near 40 cm^−1^ exhibits a high oscillator strength relative to the other modes.

**Fig. 4:**
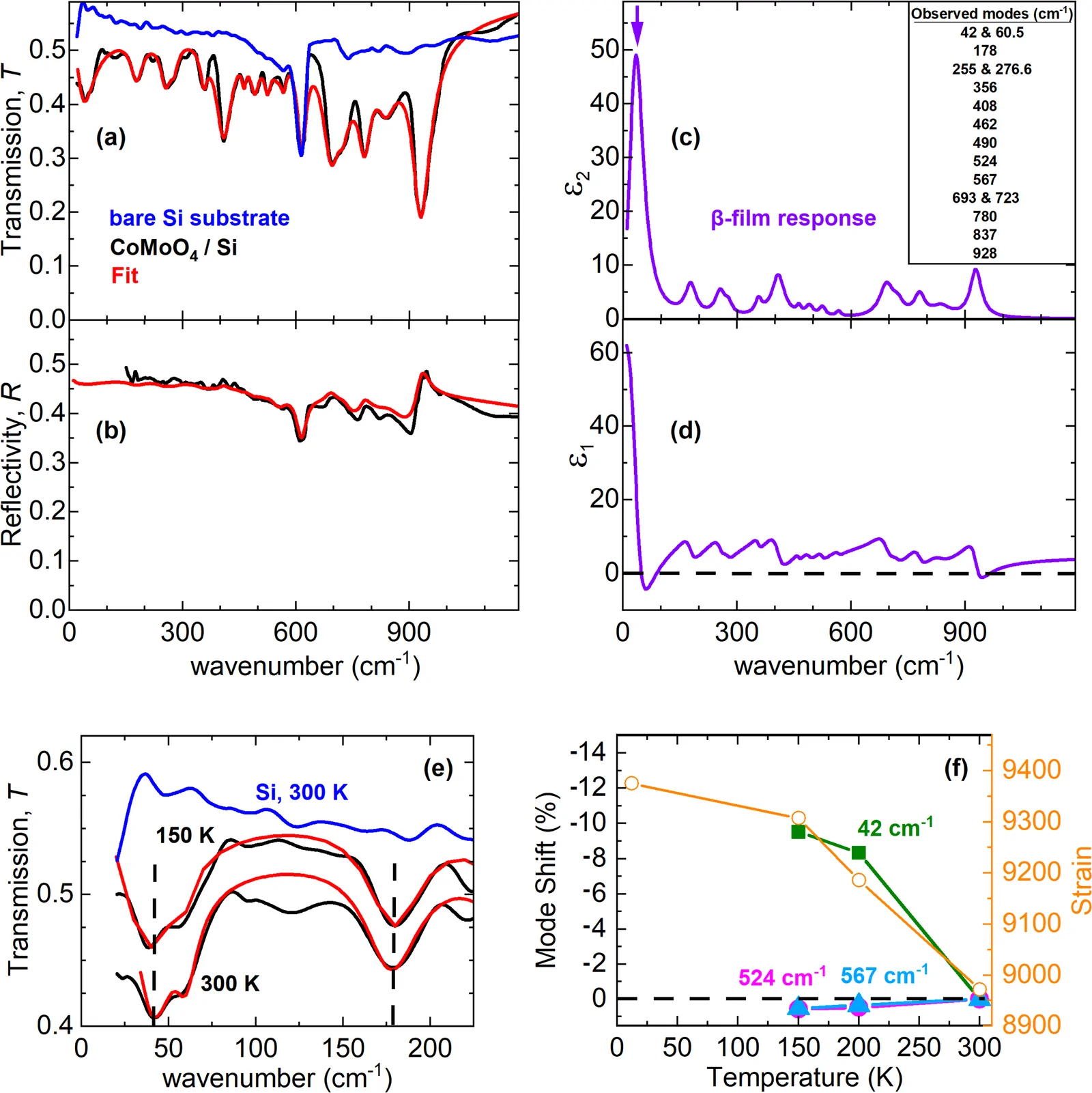
Low-energy dynamics of a 450 nm-thick β-phase CoMoO_4_ film. Room-temperature THz-to-infrared transmission and reflectivity spectra of the film grown on a silicon substrate (black), the modelled dielectric function (red) and a bare silicon substrate (blue) are shown in (a and b). The derived real and imaginary parts of the dielectric function (*ε* = *ε*_1_ + i × *ε*_2_) of the β-phase CoMoO_4_ film response are shown in (c and d), illustrating the high strength of the phonon at ∼40 cm^−1^. The inset in (c) lists 16 infrared-active modes observed at 300 K. We find three absorption bands that are best fit by two closely spaced Lorentzian functions and displayed as paired modes. The low-energy behaviour at cryogenic temperatures is shown in (e). The dashed lines highlight the 42 cm^−1^ mode that exhibits a softening with decreasing temperature and a mode at 180 cm^−1^ that exhibits a hardening. (f) Percentile mode shift of the low-energy absorption band at 42 cm^−1^ (green, square) compared to the 524 cm^−1^ (magenta, circle) and 567 cm^−1^ (cyan, triangle) bands. Between 300 K and 150 K, all modes above 178 cm^−1^ exhibit a hardening by 0.6% on average, while only the 42 cm^−1^ mode shows a softening by 9%. The observed unit cell strain, calculated from XRD measurements, is plotted for comparison (orange, open circles).

Importantly, with IR-spectroscopy techniques, we are generally not sensitive to the A_g_ and B_g_ modes, unlike the Raman scattering process. As indicated by the XRD measurements presented above, all the CoMoO_4_ films studied are in the β-phase. The crystal structure of β-CoMoO_4_ is complex, with a monoclinic *C*2/*m* space group with 8 molecules per unit cell. The Co^2+^ ions are in highly distorted octahedral sites, while two pairs of Mo^6+^ cations are in a specific tetragonal coordination in the β-phase, with *C*_2_ and *C*_s_ site symmetry.

Group theory analysis indicates a zone centre representation of

3 Γ = 19A_g_ + 17B_g_ + 14A_u_ + 19B_u_,

which implies that after subtracting the acoustic modes, there are 33 observable IR active modes, and 36 observable Raman active modes.^40,44^ According to ref. 40, 21 Raman excitations were experimentally observed, including one near 65 cm^−1^. Here, we report 16 infrared-active modes including a low-energy mode at 42 cm^−1^; a full list is displayed in the inset of Fig. 4(c). Fig. 4(e) illustrates the typical temperature dependence (between 300 K and 150 K) of the phonon modes of a 450 nm β-CoMoO_4_ film from 20 to 225 cm^−1^ wavenumbers (black). These spectra are compared with our model (red) and the ambient transmission spectra of a bare silicon substrate (blue). The spectra measured at 200 K and 150 K are nearly identical, and thus we report only the lower temperature spectra. All modes between 178 cm^−1^ and 685 cm^−1^ exhibit a hardening of about 0.6% as illustrated by the modes at 524 cm^−1^ and 567 cm^−1^, shown in Fig. 4(f), which is consistent with the observed contraction of the unit cell volume (Δ*V*/*V* ∼ 0.2%). In contrast, an anomalous softening of the strong 42 cm^−1^ absorption band is of ∼9%, which is significantly larger in magnitude than the hardening of the high energy modes. We observe a similar softening for the mode at 60.5 cm^−1^. Since the absorption band at 42 cm^−1^ is close to the low-energy end of the reliable spectral range (limit of 20 cm^−1^) due to reproducibility reasons, we measured two nominal film thicknesses of 450 nm and 200 nm and tested several fitting models to best describe the data. For instance, we have separately modelled the absorption at 42 cm^−1^ with one (at 41 cm^−1^) and then two (at 42 and 60.5 cm^−1^) Lorentzian functions; the latter two-oscillator model shows a slightly better fit on the high energy side.

As illustrated in the SI (Fig. S9) and to ensure repeatability of this anomalous low-energy spectral feature, we have measured the transmission of multiple films (with nominal thicknesses of 200 nm and 450 nm) grown on silicon substrates under ambient conditions and temperatures down to 150 K. The absorption band at 42 cm^−1^ is reproduced in all the measurements on our films and is absent in the bare substrate at any temperature (see Fig. 4(e) and S9). In addition, note that S.S Saleem reported a Raman mode at 50 cm^−1^ on a rising background, and Costa *et al.* reported a peak near 65 cm^−1^ in α-CoMoO_4_.^1,44^ Although the 42 cm^−1^ band is on the boundary of the reliable spectral data range, given the number of films studied and the Raman measurements, the evidence indicates that β-CoMoO_4_ exhibits a low energy mode at 42 cm^−1^ that softens with decreasing temperature and is likely dominated by the motion of the Co and Mo ions. A mode-softening has also been previously reported in a temperature-dependent Raman study on α- and β-phase FeMoO_4_;^39^ however, that mode is of much higher energy (∼920 cm^−1^), and the percentage softening of <1% is significantly smaller than in our case (9%), which suggests that these cases might not be directly comparable.

It is well known that β-CoMoO_4_ powder undergoes a first-order structural transition at 230 K on cooling to α-CoMoO_4_. Fig. 5 illustrates that in β-CoMoO_4_, the Mo^6+^ ions sit in a distorted tetrahedral site, while in α-CoMoO_4_, the Mo^6+^ ion moves to a distorted octahedral site. This structural transition represents a significant movement of both the Mo^6+^ and several of the coordinated oxygen ions. Given the evidence for strain in our films shown by XRD, SEM and magnetisation results discussed above, we propose that the softening of the 42 cm^−1^ mode represents the initiation of the first-order β-CoMoO_4_ to α-CoMoO_4_ transition that is suppressed by the strain and grain size in the film on the non-epitaxial silicon substrates employed. Further evidence is supplied in the following section where we discuss the high-energy electronic transitions.

**Fig. 5:**
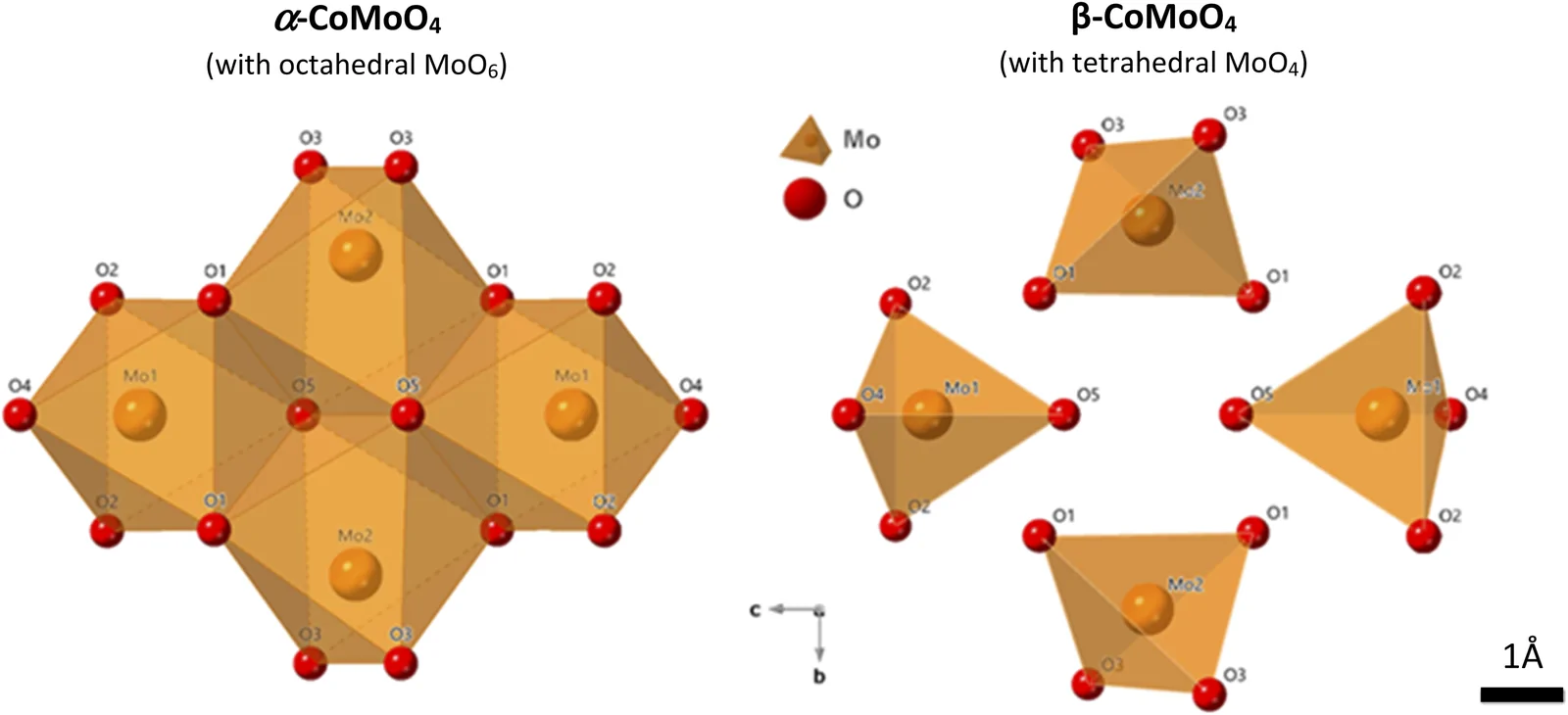
Molybdenum ion coordination in α-phase (left; octahedral MoO_6_) and β-phase (right; tetrahedral MoO_4_) CoMoO_4_.

#### Electronic transitions

Now we turn to the spectra collected between 1000 cm^−1^ and 30 000 cm^−1^ (0.12 eV to 3.7 eV) and focus on the electronic transitions. Fig. 6(a and b) displays the measured reflectivity and transmission (black curves) of a 290 nm thick β-phase CoMoO_4_ film grown on sapphire. The reflectivity and transmission were simultaneously modelled (red curves) using RefFIT, in analogy to the fitting of the phonon spectral region. In our approach, we first chose the film thickness as a variable to match the interference pattern observed. This resulted in shifts of no more than 10% from the target thickness. Next, the high-energy dielectric constant was adjusted to match the magnitudes of reflectivity and transmission. Both these adjustments were undertaken in the low-energy non-absorbing region (*i.e.*, for <4000 cm^−1^/∼0.5 eV). To describe the inter-band absorption, we included a large oscillator near the high-energy limit of the data, given the rapid decrease in transmission at around 25 000 cm^−1^. Three absorption bands at lower energy were then introduced to model the d–d band transitions for Co^2+^ using literature energies as the starting values.^3,26^

**Fig. 6:**
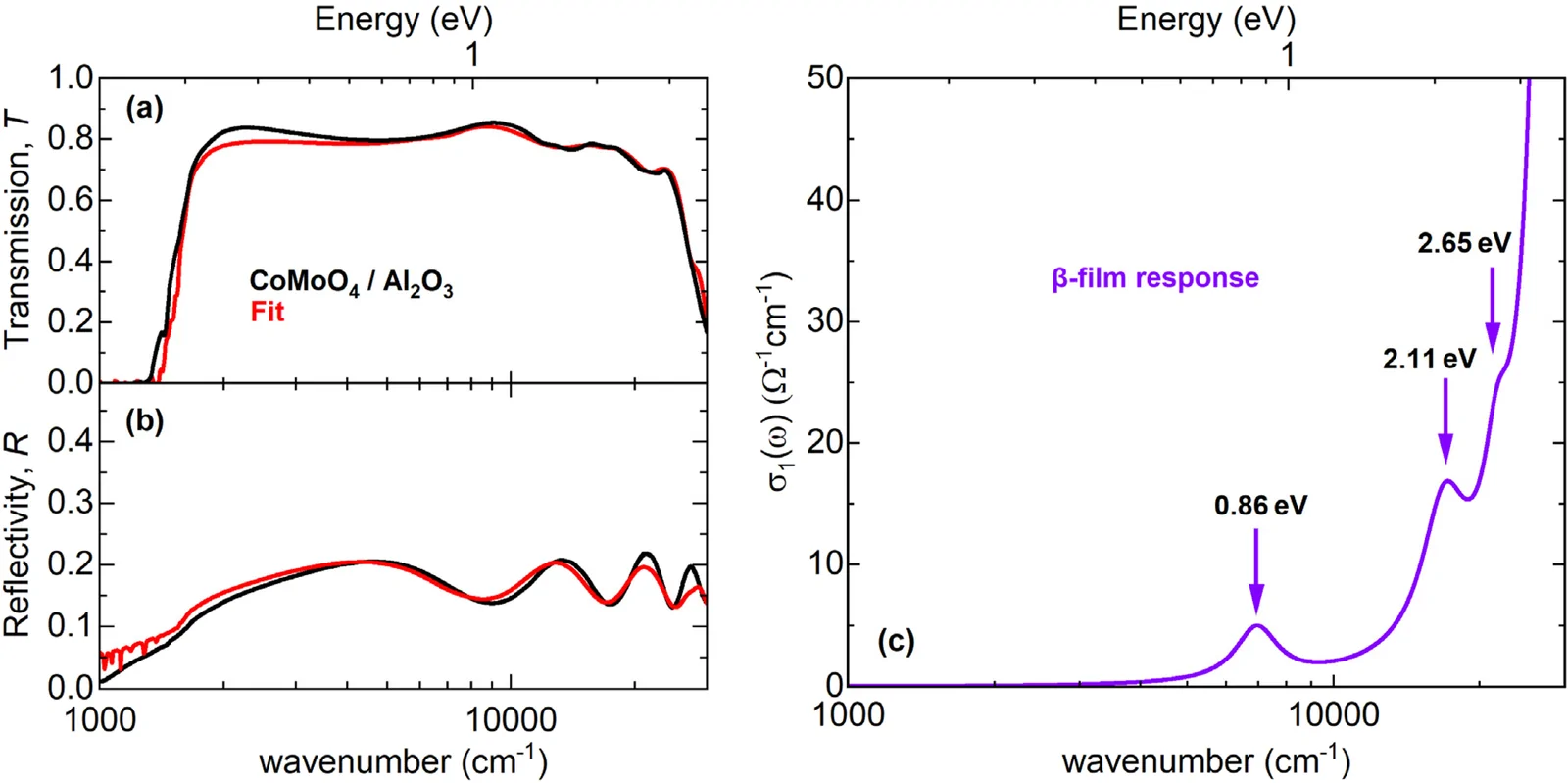
Electronic transitions – xperimental results. Experimental (black) and model (red) spectra of the high-energy transmission (a) and reflectivity (b) for a 290 nm thick β-phase CoMoO_4_ film grown on sapphire. (c) Derived optical conductivity *σ*_1_ of the β-phase CoMoO_4_ film response. The derived d–d and inter-band transitions for the β-phase CoMoO_4_ film are highlighted by arrows and listed in Table 1. As noted by Sreedhar *et al.*,^45^ the values are dependent on the crystal field environment, which is impacted by local strain.

Fig. 6(c) displays the energy-dependent real-part of the optical conductivity *σ*_1_ derived from modelling the reflectivity and transmission. Note, that the optical conductivity relates to the dielectric function *via σ*(*ω*) = −i*ω*(*ε*(*ω*) − 1)*ε*_0_, with the wavenumber *ω*, and the dielectric constant *ε*_0_. A rapid rise in the absorption occurs from about 22 000 cm^−1^ (2.7 eV) that we assign to the fundamental inter-band transition, which in the literature is often referred to as a ‘charge transfer band’, and this value is in very good agreement with reported values for β-CoMoO_4_ located in the 2.8–2.9 eV range.^1,3,5,26,44^

There exist a few band structure calculations of the transition metal molybdates in the literature; however, in analogy to ZnMoO_4_ (ref. 46) and MnMoO_4_,^47^ the valence band is dominated by oxygen p-orbitals and the conduction band by the Mo d-orbitals, while the 3d Co^2+^ orbitals are largely located in the fundamental gap and near the conduction band edge. On the lower energy side, we can identify three further narrow absorption bands (highlighted by arrows in Fig. 6(c)), which are assigned to the allowed d–d band transitions for Co^2+^ in the ^4^T_1g_(F) ground state to excited states under the octahedral crystal field of ^4^T_2g_(F), ^4^A_2g_(F), and ^4^T_1g_(P), in accordance with ref. 3. The Co^2+^ d–d band transition energies, reported in the inset of Fig. 6(c), are derived from the Lorentzian fitting model that is used to describe the optical spectra in Fig. 6(c), and are compared with the literature values for β-CoMoO_4_. The agreement in comparison to powder samples is reasonable, by taking the likely high strain state of our films into account, which will alter the crystal field of the Co^2+^ ions. This is further discussed in the following sections.

### Modified crystal field theory

To elucidate the impact of strain on the d–d spectra, we calculate the changes in Co^2+^ energy levels as the crystal field strength varies using a modified crystal field theory (MCFT).^48,49^ The results are shown in Fig. 7.

**Fig. 7:**
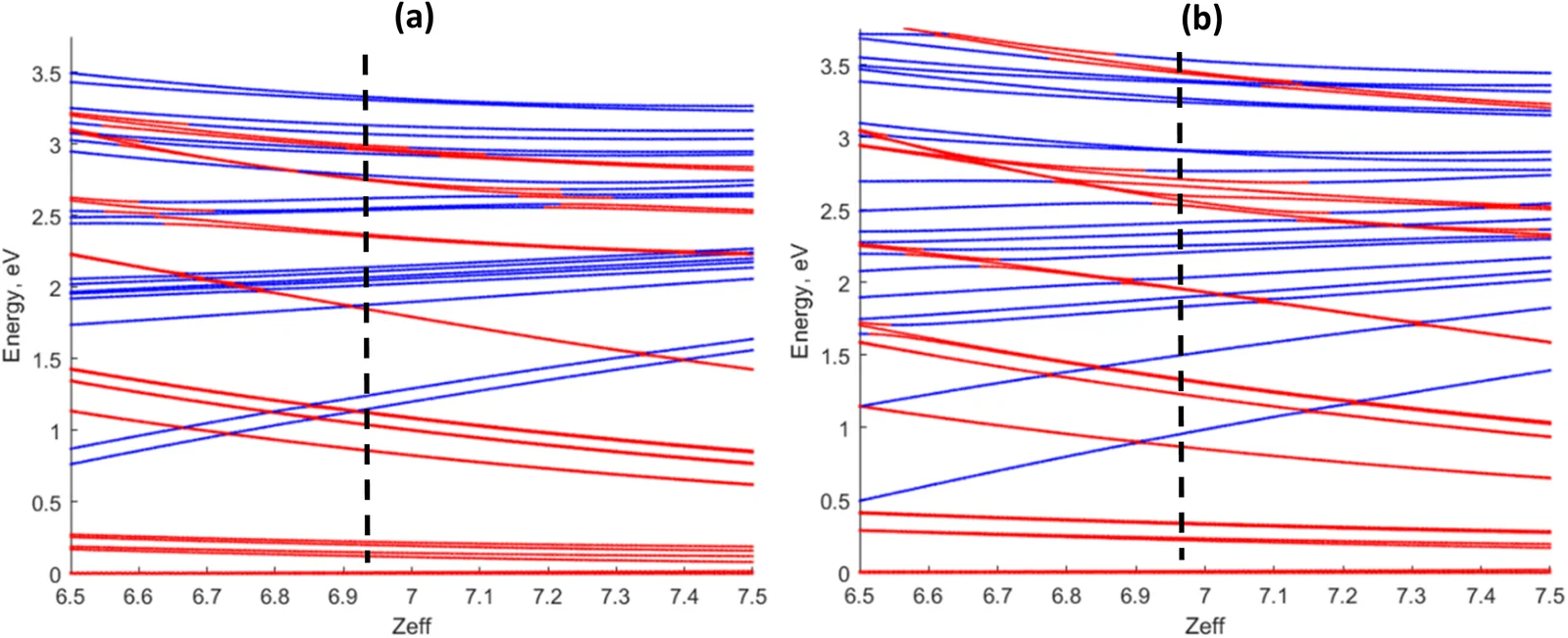
Calculated energy diagrams. The relationship between crystal field strength and energies of the Co^2+^ 3d^7^ excitations are shown for (a) the Co^2+^ ion in the 4i Wyckoff position (*C*_s_-site symmetry) and (b) the Co^2+^ ion in the 4h Wyckoff position (*C*_2_-site symmetry). The parameter *Z*_eff_, here presented in units of electron charge |*e*|, is inversely proportional to the crystal field strength. The blue lines describe the energy levels belonging to the set of terms with multiplicity *S* = 1/2; the red lines belong to the ^4^F and ^4^P terms and show a gradual increase with higher crystal field strength. Vertical dashed lines denote *Z*_eff_ = 6.925 in (a) and *Z*_eff_ = 6.975 in (b) at which the theoretical energy levels coincide well with experimentally observed bands at 0.86, 2.11 and 2.65 eV.

In the MCFT approach, the crystal field strength is parameterised by the effective nuclear charge of the metal ion-*Z*_eff_, which decreases under the influence of the ligand's electron clouds compared to its value for the free ion. Thus, a decrease in the effective nuclear charge describes an increase in the crystal field strength. The MCFT input parameters are the valence state, the exact positions of the ligands in the crystal unit cell, as well as the position of the cation. The spin–orbit coupling is also accounted for.^48^ We used the crystallographic data from the SI of Robertson *et al.*^3^ and the data for the lattice constants of our β-phase thin film at 12 K from Table S1.

The energy diagrams for both positions of Co^2+^ ions in the β-phase of CoMoO_4_ film are shown in Fig. 7. We demonstrate 38 Kramers doublets out of 60 of the Co^2+^ 3d^7^ configuration, with energy below 3.75 eV in the range 6.5|*e*| < *Z*_eff_ < 7.5|*e*|, with *e* being the electron charge. Note that, for the free ion Co^2+^*Z*_eff_ = 8|*e*|, as reported in ref. 50. The energy levels originating from the ^4^F and ^4^P terms of the free ion (*i.e.* from the terms with the highest multiplicity 2*S* + 1 = 4) are shown by red-coloured lines. It is expected that the most pronounced transitions, so-called “spin-allowed transitions”, occur between the levels with the same multiplicity. The low site symmetry *C*_2_ and *C*_s_ at both Co^2+^ positions results in a complete splitting of the levels initially assigned to a purely octahedral ligand environment. Note, that our calculations also suggest a splitting of the observed low-energy mode centred at 40 cm^−1^ when being exposed to an external magnetic field, which remains a subject for future research.

Table 1 shows the experimentally observed and the calculated transition energies (using the experimentally determined lattice parameters for our films) compared with transition energies calculated using lattice parameters for the powder.^3^ As noted by Sreedhar *et al.*,^45^ the transition energies are dependent on the crystal field environment, which is impacted by local strain. The experimentally obtained values for the hardening of the d–d band energies in our films are in good agreement with literature values as shown, which provides further evidence of a strained state of the β-phase in our CoMoO_4_ films.

**Table 1:** Experimentally and computationally derived d–d and experimentally determined inter-band transition energies for a β-phase CoMoO_4_ film, in comparison to computational and literature values for β-phase CoMoO_4_ powder^1,3,5,26^

Electronic transition energies in β-CoMoO_4_ [eV]				
Transition	Thin film		Powder	
	Experiment	Calculation	Experiment	Calculation
^4^T_1g_(F) → ^4^T_2g_(F)	0.86	0.86	0.84	0.83
^4^T_1g_(F) → ^4^A_2g_(F)	2.11	2.12	1.76	1.9
^4^T_1g_(F) → ^4^T_1g_(P)	2.65	2.7	2.13	2.1
O^2−^ → Mo^6+^	2.70	—	2.8–2.9	—

As illustrated in the calculated energy diagrams in Fig. 7 and listed in Table 1, the energy of the spin-allowed d–d transitions exhibits an increase with increasing crystal field strength. In our case, there are two almost degenerated Kramers doublets with energies between 0.5 and 1 eV where a spin-allowed d–d transition may occur, *i.e.* the calculated energies of the two Co^2+^ sites are sufficiently separated in energy to clearly assign the measured absorption bands to individual cobalt ions in the two different lattice positions. The energy of these d–d transitions can nonetheless serve as a characteristic marker of the crystal field strength in the CoO_6_-cages. Note that the results of the MCFT calculations can be considered as a suitable guide for a qualitative explanation of the electronic spectra of Co ions under a change of the crystal field strength in the CoMoO_4_ crystal. They may also provide a quantitative answer in the energy range far away from the ligand p-orbital energies, *i.e.* below the energy of the charge transfer transition band. In our case, this range is less than 3 eV. Moreover, our method for analysing the thin films is different from that used on diffuse reflectance measurements of powders where the narrow absorption bands are located on the rising inter-band absorption region, which is an issue that becomes more significant at higher energies where the discrepancy is at the maximum.

## Summary

Thin films of β-CoMoO_4_ were successfully synthesised on silicon and sapphire substrates, and their structural, magnetic, and optical properties were investigated. Temperature-dependent X-ray diffraction down to 12 K confirms that the films do not undergo the β → α transition observed in powders. Our multimodal characterisation approach (XRD, SEM, magnetisation, optical spectroscopy, and supported by MCFT calculations) allows provision of strong evidence that strain is a dominating factor in our films that governs the suppression of this thermochromic phase transition. Magnetic measurements down to 2.2 K confirm an antiferromagnetic transition near 10 K, consistent with our previous reports on powder samples, but with no magnetic hysteresis observed in the thin films. A field-induced transition at ±2.2 T is seen below the Néel temperature, with a sharp magnetisation increase that is characteristic of a spin–flip transition, indicating strong magneto-crystalline anisotropy. We argue that due to the sharpness of this transition and as suggested by our calculations, we have classified it as a spin–flip transition, which is in contrast to what was observed in other molybdate systems. Room-temperature optical spectroscopy (terahertz to visible/UV) allows the identification of interband transitions and three Co^2+^ d–d excitations, in good agreement with the MCFT calculations. We also identify 16 infrared-active phonons. Upon cooling down to 150 K, being across the 1st order transition of β to α-phase in powder samples, we observe that the higher-energy phonon modes (178–680 cm^−1^) exhibit hardening which is consistent with lattice contraction, while the lowest-energy mode at 42 cm^−1^ softens, dominated by cation motion. This anomalous behaviour is attributed to incipient motion of Mo^6+^ ions from their tetrahedral position in the β-phase to an octahedral site in the α-phase, which is suppressed in the thin film due to microstrain. MCFT calculations support a strained state in the films and suggest a splitting of this mode when exposed to an applied magnetic field, which remains a subject of future research. These results demonstrate that strain stabilises the β-phase and modifies both lattice dynamics and electronic structure, providing a route to control functional properties in thermochromic oxides and relevant for the design of optical sensing devices.

## Author contributions

KVK, KHJH, RGB, DA and FL conducted the optical THz-Vis-UV experiments and analysis, with input from YGP. SVC and JW grew the samples and performed the magnetization and room-temperature X-ray diffraction (XRD) measurements and analysis. The modified crystal field theory calculations were done by YGP and SMO. EL and MG carried out the temperature-dependent XRD experiments. The scanning electron microscopy (SEM), Rutherford backscattering spectrometry (RBS) and X-ray photoelectron spectroscopy (XPS) experiments and analysis were done by PPM and SS. FL, SVC and RGB conceived and led the project. RGB, YGP, SVC and FL wrote the manuscript with input from all authors. All authors discussed the results.

## Conflicts of interest

There are no conflicts to declare.

## Supplementary Material

SC-OLF-D6SC04298H-s001

## Data Availability

The data supporting this article have been included as part of the supplementary information (SI). Supplementary information is available. See DOI: https://doi.org/10.1039/d6sc04298h.
