# Supplementary material for: Strain-induced suppression of thermochromism in divalent cobalt molybdate thin films
Source: Chem Sci. 2026 Oct 16. Online ahead of print. doi: 10.1039/d6sc04298h (PMC13673700; doi:10.1039/d6sc04298h)
Supplement: Supplementary file 1 [file SC-OLF-D6SC04298H-s001.pdf]

Supplementary Information (SI) for Chemical Science.
This journal is © The Royal Society of Chemistry

Supplementary Materials

Strain-induced suppression of the thermochromic transition in divalent cobalt molybdate thin films

Kiri Van Koughnet[1], Joey Williamson[1], Kane H. J. Hill[2,3], Robert G. Buckley[1,4], Yurii G. Pashkevich[5,6], Sergej M. Orel[6], Eric Lebraud[7], Manuel Gaudon[7], Peter P. Murmu[8], Sarah Spenser[1], Dominique Appadoo[9], Shen V. Chong[1,4], Fryderyk Lyzwa[2,3,4]

[1]Robinson Research Institute, Victoria University of Wellington, P.O. Box 33—436, Lower Hutt 5046, New Zealand
[2]Department of Physics, Photon Factory, University of Auckland, 38 Princes Street, Auckland 1010, New Zealand
[3]Te Whai Ao Dodd-Walls Centre for Photonic and Quantum Technologies, New Zealand
[4]MacDiarmid Institute for Advanced Materials and Nanotechnology, P.O. Box 600, Wellington 6140, New Zealand
[5]Department of Physics and Fribourg Center for Nanomaterials, Chemin du Musée 3, University of Fribourg, Fribourg, Switzerland
[6]O.O. Galkin Donetsk Institute for Physics and Engineering NAS of Ukraine, 03028 Kyiv, Ukraine
[7]Univ. Bordeaux, CNRS, Bordeaux INP, ICMCB, UMR5026, 87 avenue du Dr. A. Schweitzer, Pessac, F-33608, France
[8]National Isotope Centre, Earth Sciences New Zealand, 30 Gracefield Rd, PO Box 30368, Lower Hutt 5010, New Zealand
[9]THz/Far-IR Beamline, Australian Synchrotron, 800 Blackburn Rd, Clayton, VIC, 3168 Australia

THz/Infrared transmission data: Raw and noise-processed spectra

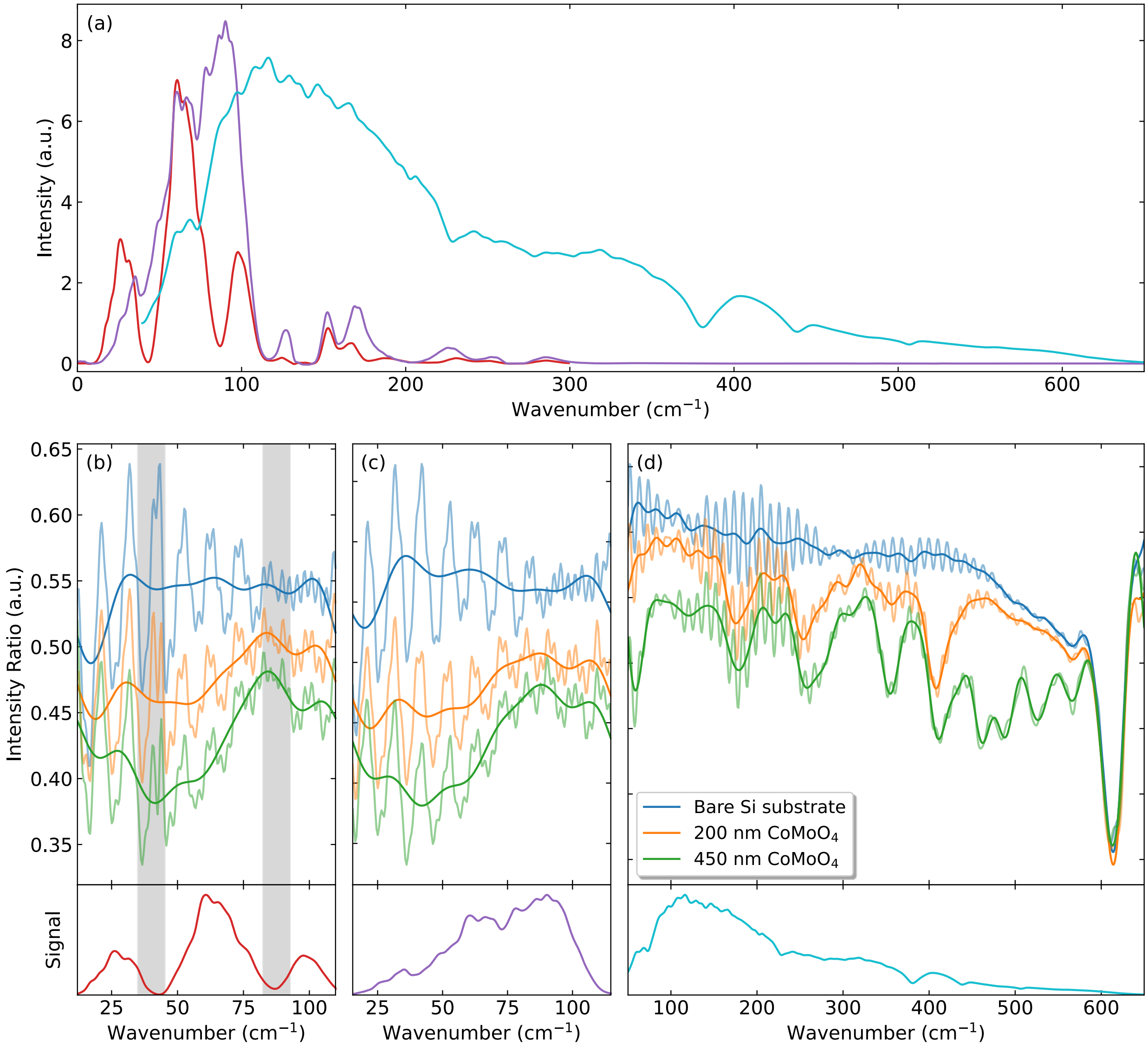

Figure S1. (a) Raw transmission spectra of a β-$CoMoO_4$ film grown on silicon substrate, as taken at the Australian Synchrotron THz spectroscopy beamline. To cover the spectral range from ca. 20 - 600 cm^{-1}, we used different combinations of cold filters and beamsplitters made of mylar (with thicknesses of 6 µm and 75 µm), leading to three distinct spectral regions as outlined in (b), (c) and (d). The oscillatory noise signal contribution visible in the intensity ratio is assumed to originate from multiple reflections of the silicon substrate as they are absent with no sample being installed; the spectra were corrected by applying a low-pass filter to the inverse Fourier transform of each intensity ratio. Importantly, we observe a significant signal intensity down to at least 20 cm^{-1} as seen in (b). The grey-coloured bars represent the spectral regions with low signal intensity, that are complimented with the signal obtained from another cold filter & beamsplitter combination displayed in (c).

Temperature-dependent x-ray diffraction (XRD)

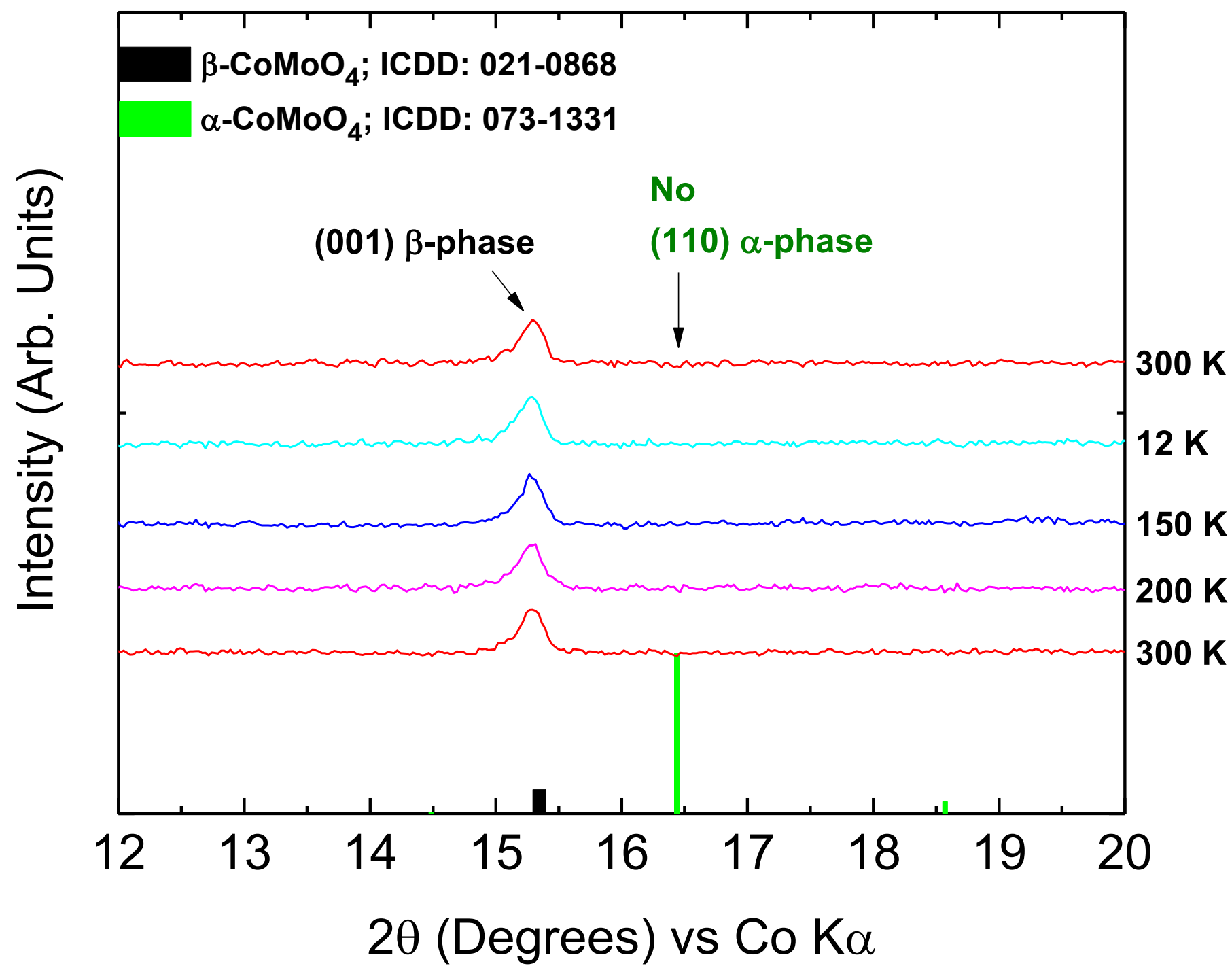

Figure S2. Temperature-dependent X-ray diffraction (XRD) patterns of a $CoMoO_4$ thin film without a pre-existing α-phase. The reference patterns of α- and β-$CoMoO_4$ are based on the International Centre for Diffraction Data (ICDD). The sample was cooled from 300 K (bottom curve) to 12 K then back to 300 K (top curve).

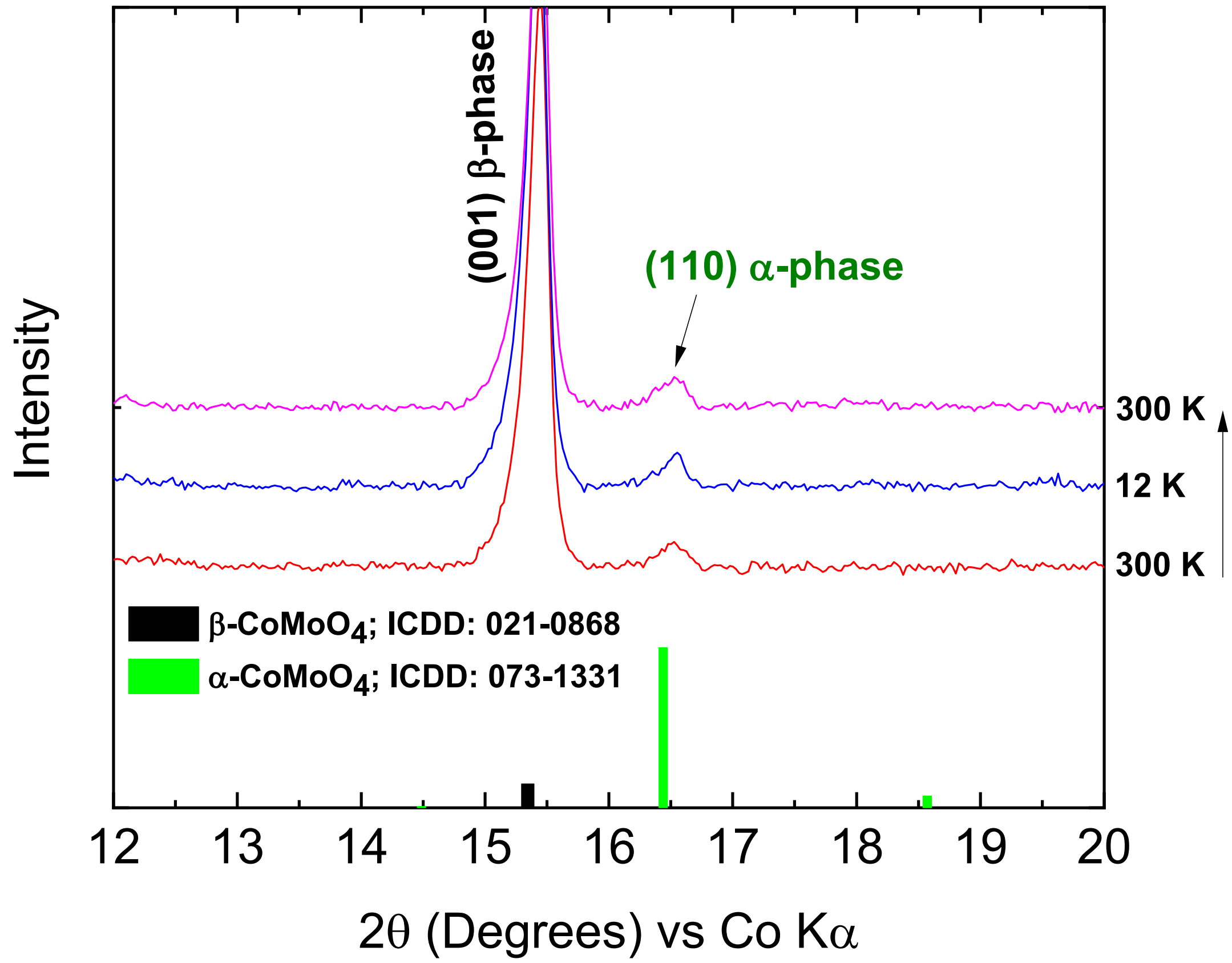

Figure S3. Temperature-dependent X-ray diffraction (XRD) patterns of a $CoMoO_4$ thin film with a pre-existing α-phase. The vertical arrow indicates the direction of cooling-and-heating and measurement sequence.

	300 K (before cooling)	200 K	150 K	12 K	300 K (after warming)
**Lattice Parameters:**					
a (Å)	10.239(5)	10.238(7)	10.237(6)	10.237(6)	10.238(5)
b (Å)	9.280(1)	9.277(2)	9.270(2)	9.272(2)	9.282(1)
c (Å)	7.025(3)	7.025(4)	7.023(3)	7.022(3)	7.025(3)
β (°)	107.89(1)	106.91(1)	106.90(1)	106.92(1)	106.90(1)
Vol. (Å^3)	638.7(2)	638.4(2)	637.7(2)	637.7(2)	638.7(2)
Strain	8972.1	9186.1	9306.8	9375.3	9025.3
Texture Ind	4.5(1)	5.5(2)	6.5(2)	5.1(2)	4.6(1)
wR	2.12	1.83	1.91	2.06	2.07

Table S1. Lattice parameters of β-$CoMoO_4$ thin film sample at different temperatures.

X-ray photoelectron spectroscopy (XPS)

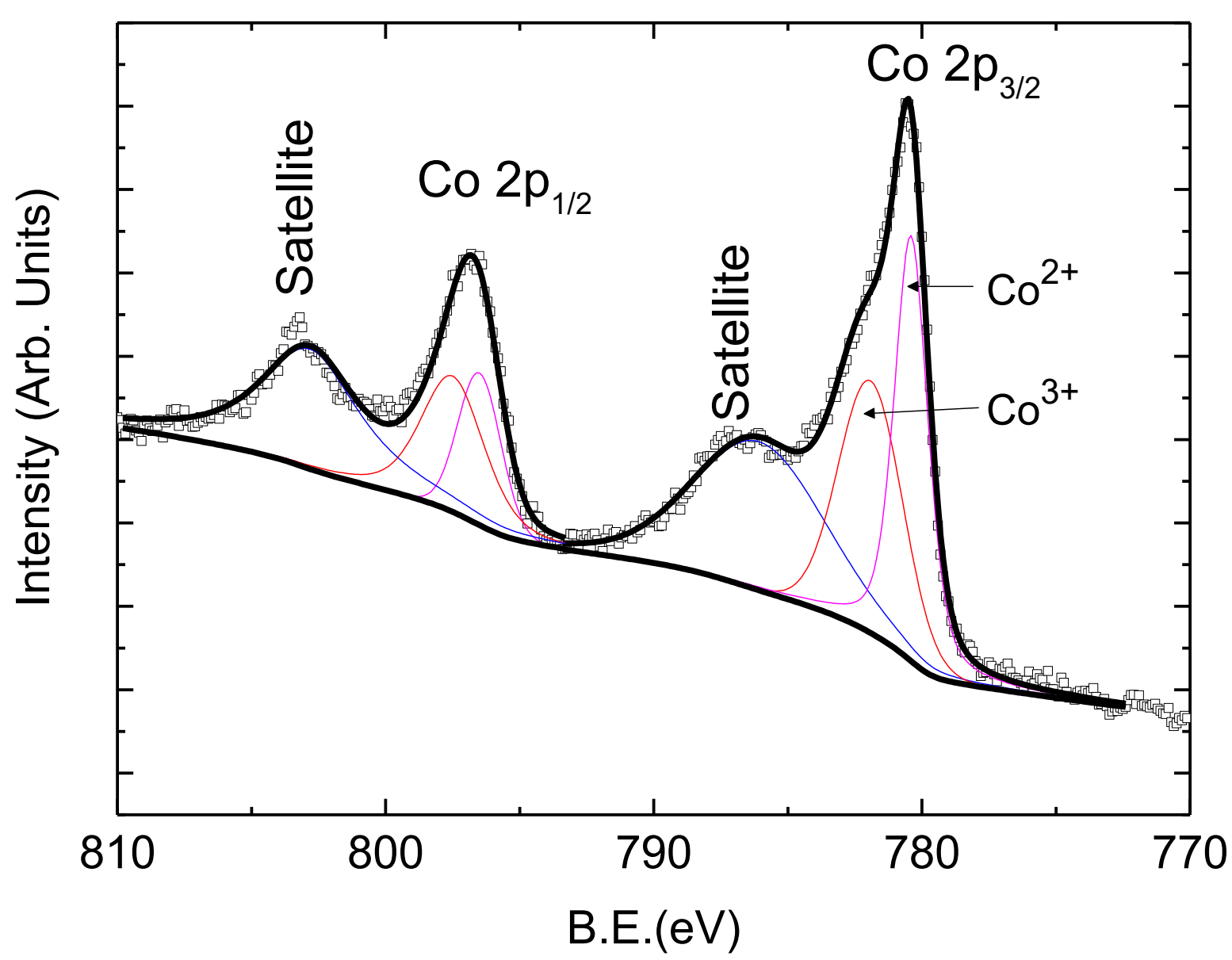

Figure S4. XPS Co 2p core level of a β-$CoMoO_4$ thin film measured at 300 K.

Grazing angle XRD of a $CoMoO_4$ thin film annealed under reducing environment

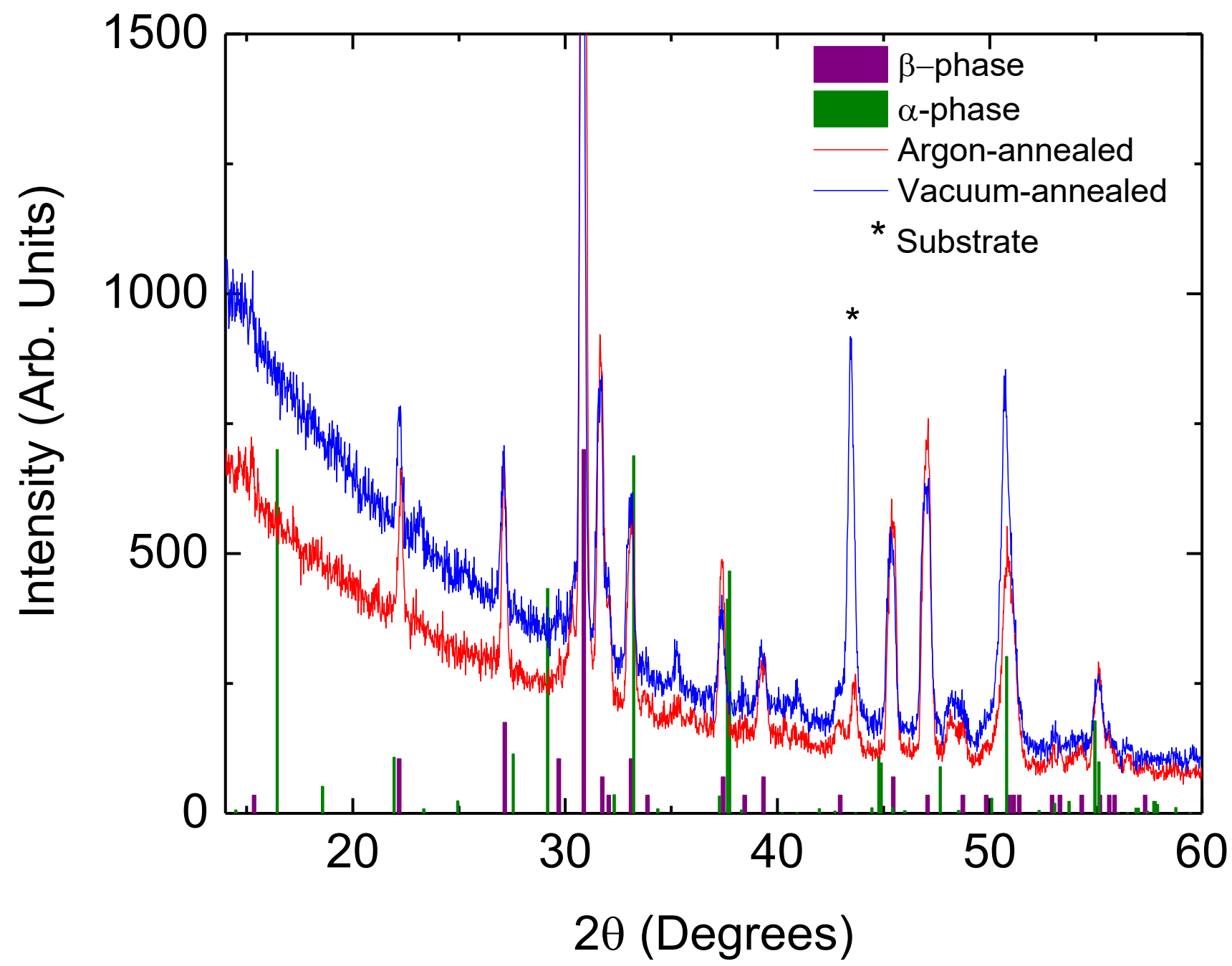

Figure S5. Grazing angle XRD of a $CoMoO_4$ film annealed at 600 °C under argon gas flow (red) and separately under vacuum (blue). The XRD measurements were carried out after the film had been cooled down to 2.2 K and subsequently warmed back to room temperature. No evidence of α-$CoMoO_4$ is observed in either film, as this phase would exhibit a prominent reflection at 16.4° = 2θ.

Temperature-dependent magnetization data, and sample annealing effect

First under argon gas flow, then in a vacuum of ~1×10^{-3} Torr: The sample did not display a hysteresis around the spin transition temperature (2.2 Tesla) that could be associated with the presence of α-$CoMoO_4$, as seen in our previous study [S1]. Finally, the film was subsequently annealed under O_2 gas flow: The change in the magnetisation at high fields was minimal, and no hysteresis was observed around the spin flip transition field; the slight reduction in magnetisation is negligible small. These results further confirm the absence of a β to α phase transition, and the presence of Co^{3+} species on the film surface has no effect on the phase transition behaviour of $CoMoO_4$ thin films.

The abrupt low temperature magnetization changes at relatively high magnetic field of 2.2 T with a relatively low T_N=10 K points to anomalously large magnetic anisotropy in the β-$CoMoO_4$ films. In the case of an Ising-like antiferromagnets (AFM), we do not expect the spin-flop transition to appear in the applied magnetic field along the anisotropy direction (see below), but only the spin-flip one. The spin-flip transition occurs if the magnitude of the critical magnetic field, H_c, reaches the effective antiferromagnetic exchange field.

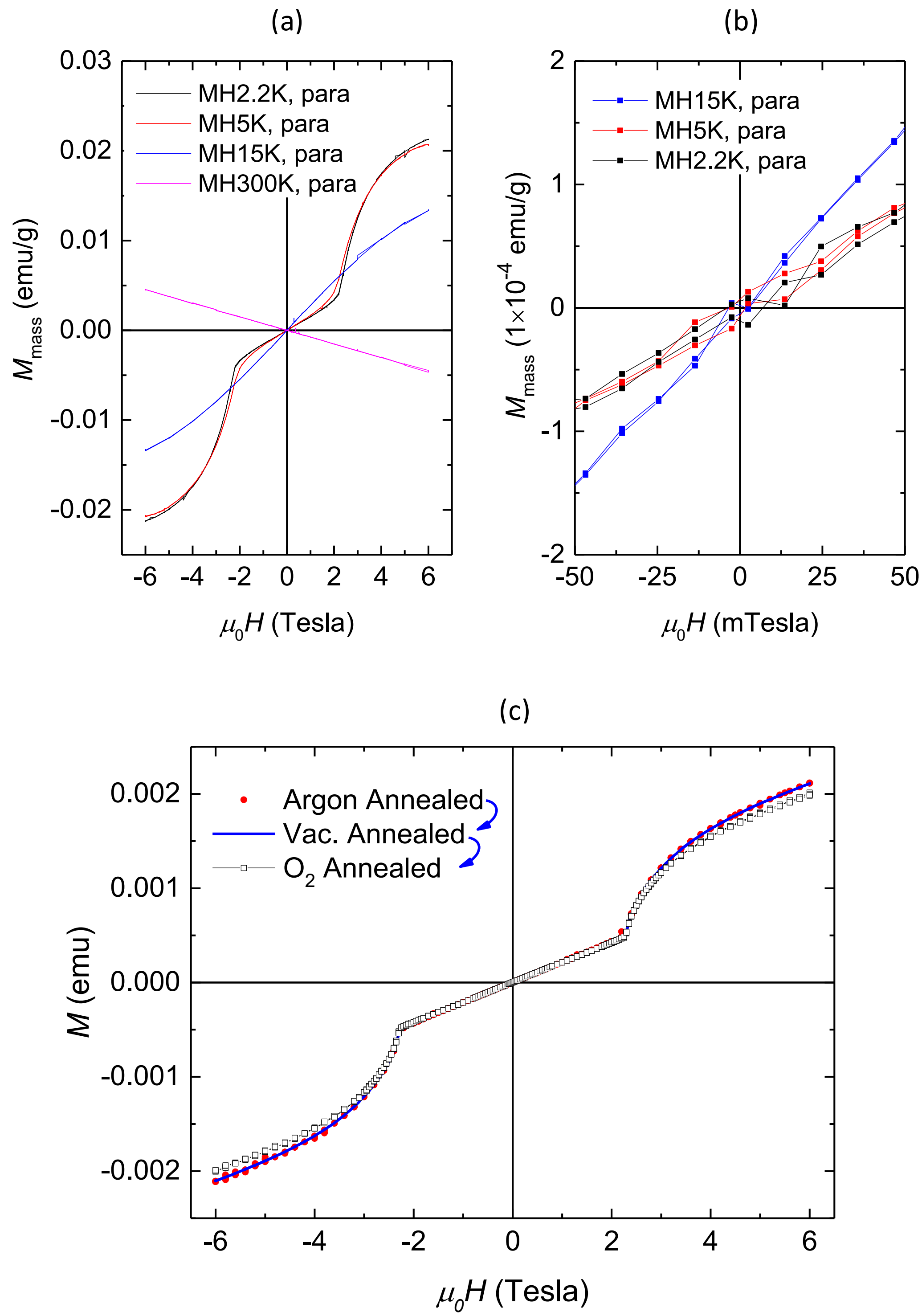

Figure S6. (a) Temperature-dependent field-loop magnetisation of a pure β-$CoMoO_4$ thin film measured with the surface parallel (para) to the applied magnetic field. (b) The field loops below 10 K show no hysteresis in the low field regions (right). (c) Annealing effect: Magnetisation field-loops at 2.2 K of a β-$CoMoO_4$ film (450 nm nominal thickness) that has been annealed post-growth ex-situ at 600 °C and under various conditions.

The field dependencies for the spin-flip transition in polycrystalline samples has specific form for the fields higher than H_c. If the easy axis is directed at an angle θ to the magnetic field, then the spin-flip transition occurs at higher field $H_c(\theta) = H_c(0)/\cos\theta$. Thus, for the case of the polycrystalline sample the spin-flip transition must arise at $H_c(0)$ and develops towards higher field. Under averaging on all randomly oriented grains and at zero temperature, the magnetisation $M(H,0)$ has the form:

$$M(H,0) = \frac{Ms(0)}{2}\left(1 - \frac{H_c(0)^2}{H^2}\right) \text{ for } H > Hc(0) \text{ and } M(H,0) = 0 \text{ for } H < Hc(0) \qquad \text{(S1)}$$

Here, $Ms(0)$ is the saturated magnetization of a grain at zero temperature and at θ=0. $Hc(0)$ is a magnitude of spin-flip field at zero temperature for a unique grain.

At nonzero temperatures, the magnetisation curve of the polycrystalline sample with Ising-like AFM order can also be roughly divided into two regions. The first one present linear dependence $M(H,T) = \chi(T)H$ which arise due to the field-induced change in the absolute magnitude of the up and down sublattice magnetisations, so-called paraprocess. It should be noted that in this region of the field and for low temperatures, the contribution to the magnetisation from the simple bending of the opposite magnetic moments is negligible.

The second one has the same form as Eq. S1:

$$M(H,T) = \frac{Ms(T)}{2}\left(1 - \frac{H_c(T)^2}{H^2}\right) \qquad \text{(S2)}$$

Here, $Ms(T)$ and $H_c(T)$ are the saturated magnetisation and spin-flip field at nonzero temperature for a unique grain. In Fig.S7, we apply Eq.S2 to the fitting of the nonlinear parts of the $M(H,T)$ curves of Fig.S5. The best results we obtained are $Ms(2.2K) = 0.0486\,\frac{emu}{g}$ and $H_c(2.2K) = 2.09\,T$ for 2.2 K and

$Ms(5K) = 0.0466\,\frac{emu}{g}$ and $H_c(5K) = 2.0\,T$ for 5 K. As expected, the value $Ms(T)$ and $H_c(T)$ decrease with increasing temperature.

Such unique behaviour as described by Eq. S2 on the experimental magnetisation curves (see Fig. S7) of our polycrystalline sample clearly indicates the existence of an Ising-like AFM structure in our samples.

Modified crystal field (MCFT) calculations of *g*-factors for the first Kramers doublets of Co1 and Co2 ions yield highly anisotropic *g*-factors (see Eq. S3). In these calculations, we have used the lattice constants of the strained film from Table S1 and the restored effective cobalt nuclear charges, Z_{eff}, from the *d*-*d* spectra shown in Fig.7.

$$g(Co1, \text{Zeff} = 6.925) = \begin{pmatrix} 0.7068 & -0.1128 & -0.3436 \\ -0.1128 & 7.0657 & 0.2336 \\ -0.3436 & 0.2336 & 2.1142 \end{pmatrix}$$

$$g(Co2, \text{Zeff} = 6.975) = \begin{pmatrix} 0.1830 & 0.0014 & -0.0011 \\ 0.0014 & 7.6950 & -0.0390 \\ -0.0011 & -0.0390 & 0.2592 \end{pmatrix} \qquad \text{(S3)}$$

Such strong *g*-factor anisotropy is typical for the non-Kramers ions and implies an Ising type antiferromagnetic order with the easy axis provided by the maximal *g*-factor component, whereas contribution from other components can be neglected. Interestingly, the calculated *g*-factors for both cobalt ions in the normal unstrained β-phase do not exhibit such strong anisotropy (not shown).

According to Eq.S3, the corresponding Ising-type antiferromagnetic magnetic order in the strained β-$CoMoO_4$ film should be collinear and should be realized mainly along the *b*-axis for both Co sites (the simplest antiferromagnetic order is shown in Fig. S8 below). It should be noted that the antiferromagnetic order parameters for the Co1 and Co2 sublattices, if directed along the *b*-axis, are not related to each other for reasons of symmetry [S2]. Here we account for the same space group and the same structural units for $FeMoO_4$ and $CoMoO_4$.

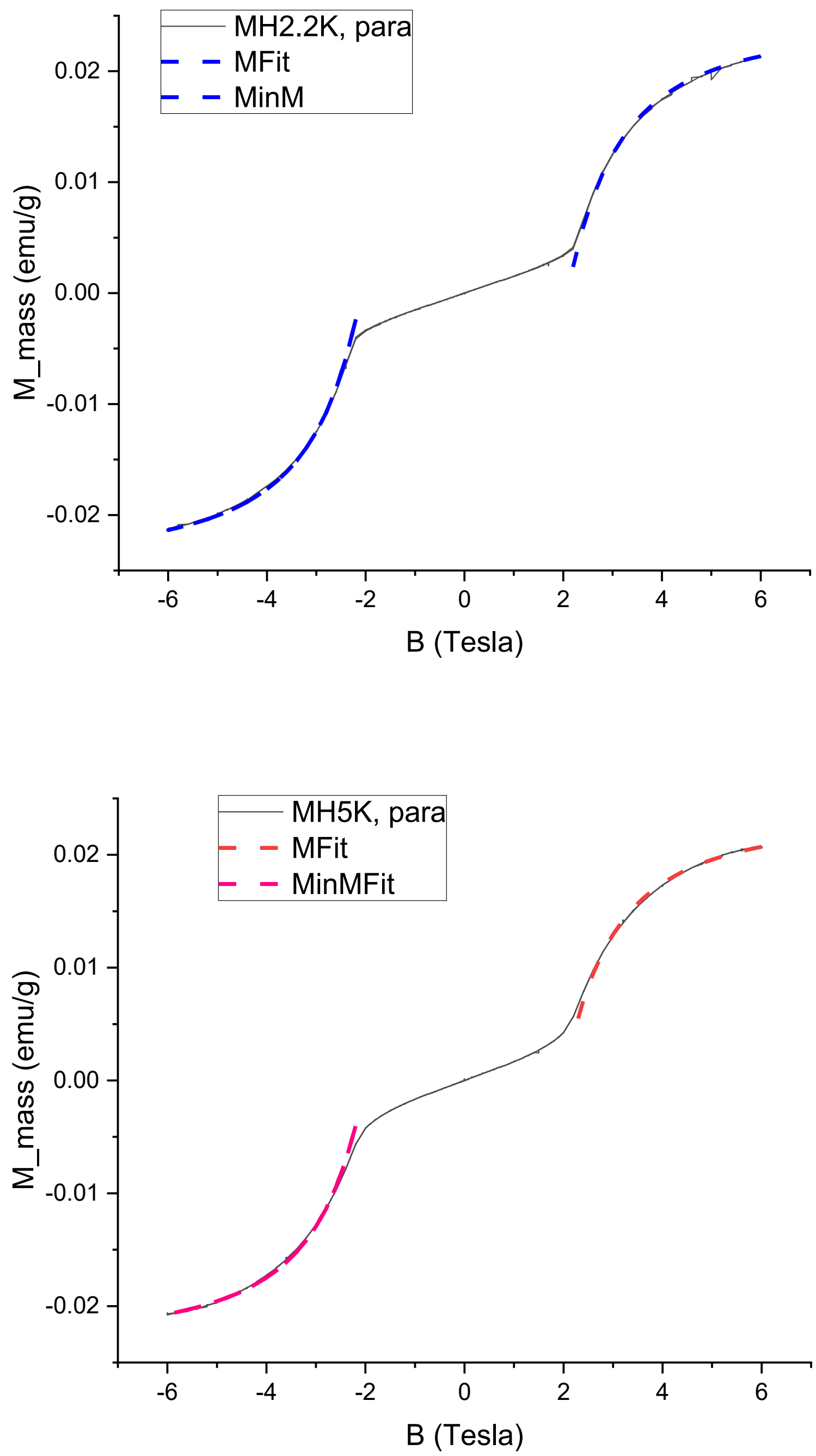

Figure S7. Partial fit of the magnetization curves of β-$CoMoO_4$ thin film in the nonlinear region $M(H)$ using Eq. S2 derived for a spin flip transition in polycrystalline samples.

Possible magnetic structure of the strained film

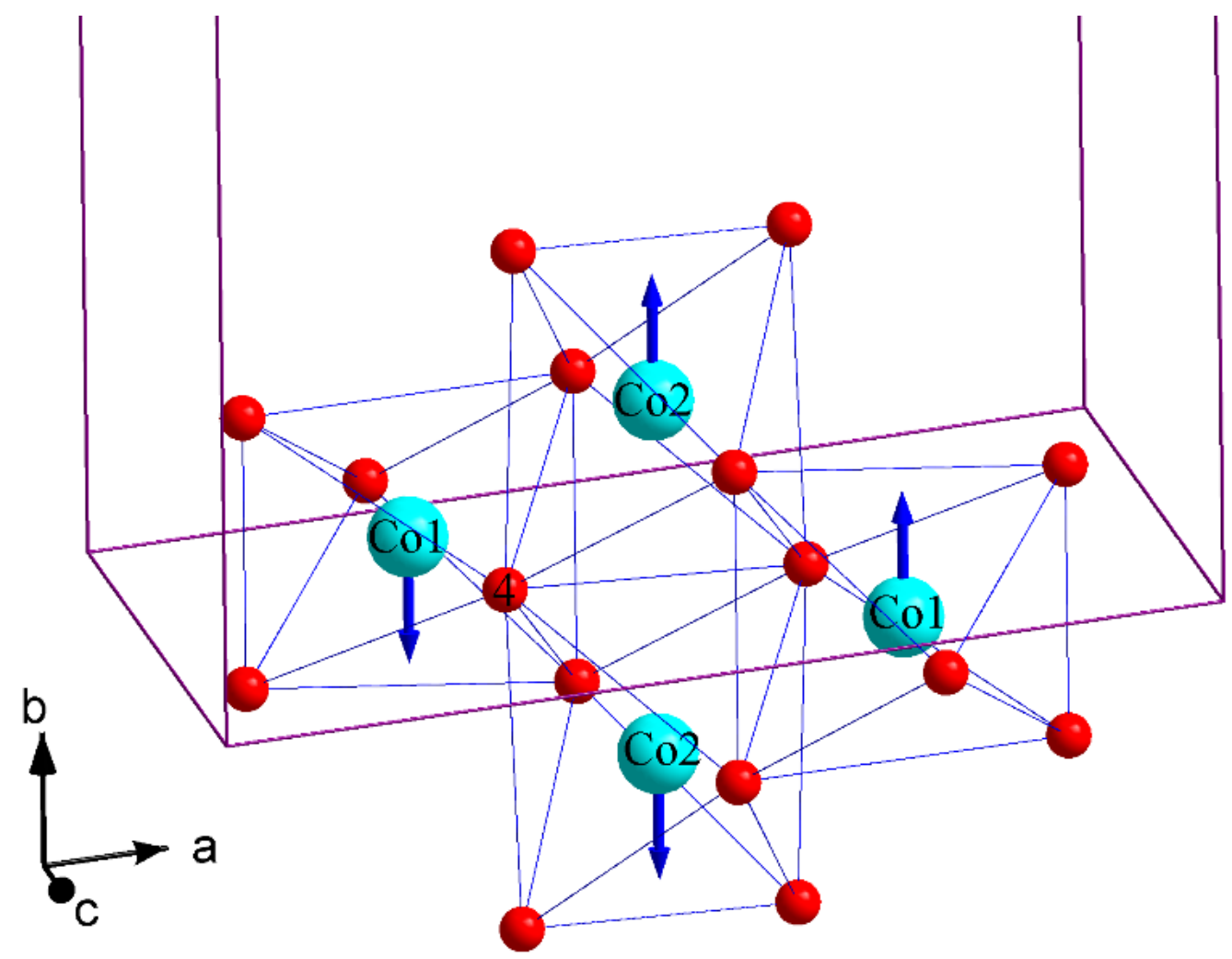

Figure S8. Possible magnetic structure of the strained β-phase of $CoMoO_4$ film. The only Co tetramer, so called Co_4O_{16} butterfly motif, is shown. The oxygen ions shown in red, blue arrows denote magnetic moments.

Temperature-dependent THz/Infrared spectroscopy

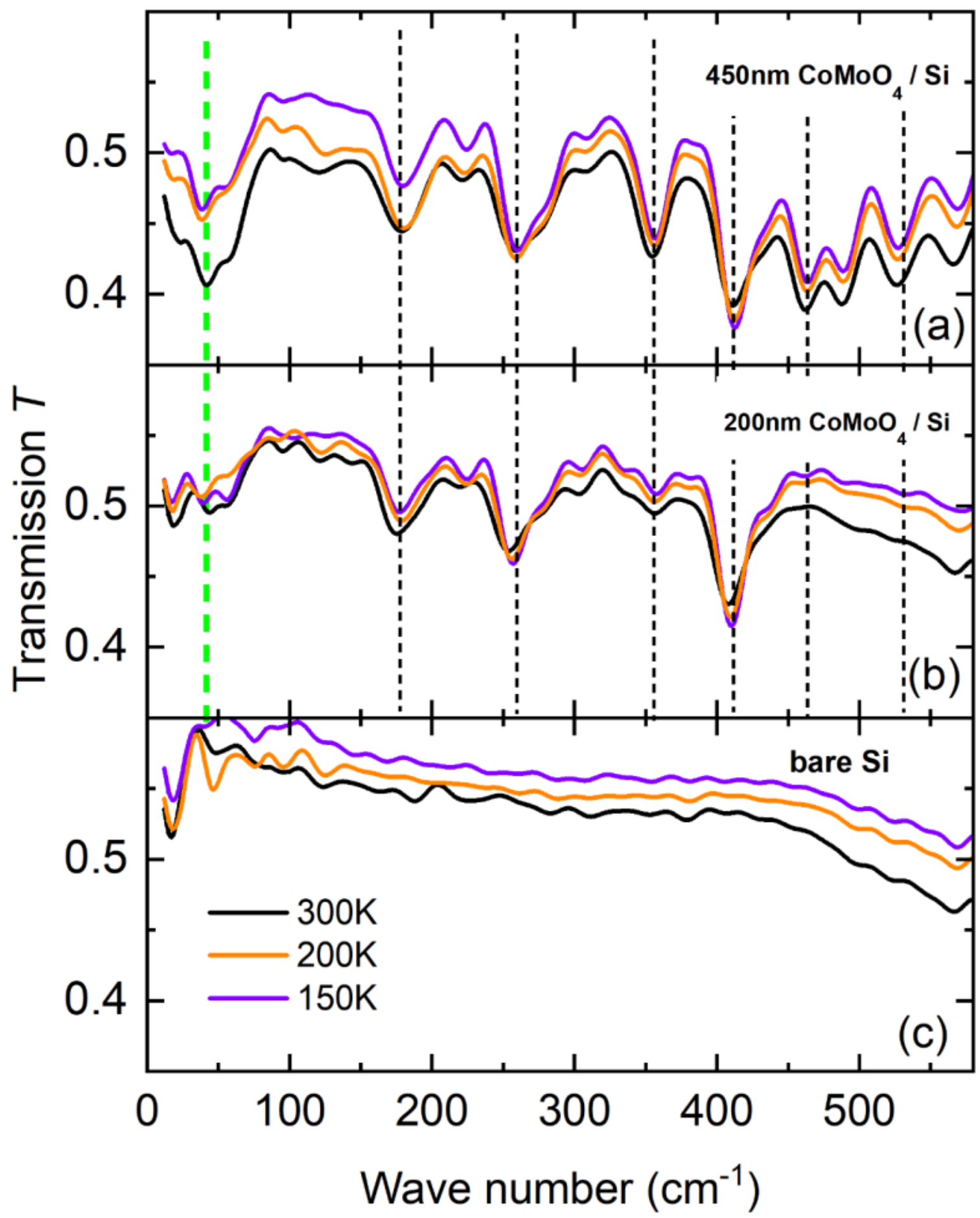

Figure S9. Temperature-dependent Terahertz/Far-infrared transmission at 300 K, 200 K and 150 K for two β-$CoMoO_4$ films on silicon substrates with different target thicknesses of 450 nm (a) and 200 nm (b). Of strong interest is the low-energy absorption at 40 cm^{-1} that softens with decreasing temperature observed in both films at all temperatures. As is illustrated, the modes at higher energy harden. These spectra are compared in the bottom panel (c) with the spectra of a bare silicon substrate (0.5 mm) at the same temperatures which do not exhibit a band at 40 cm^{-1}.

Modified Crystal Field Theory (MCFT)

Cobalt ions in the α- and β-phase of $CoMoO_4$ occupy two Wyckoff positions: Co(1) – 4i with the site symmetry group C_s containing elements (e, m_y), and Co(2) – 4h with the site symmetry group C_2 containing elements (e, 2_y). In both positions, the symmetry of the highly distorted octahedral oxygen cages does not include space inversion. Both site groups contain only two dipole active irreducible representations: C_s -A'(x,z); A'(y) and C_2 – A(y); B(x,z) (notations are taken from Bilbao server). Thus, all 60 Kramers doublets (120 energy levels) contained in the free ion terms of the $3d^7$ configuration are completely split by the mechanisms of spin-orbit coupling and low symmetry. This feature makes the attribution of the *d-d* spectra absorption based on an ideal octahedral crystal field environment rather questionable.

On the other hand, the low symmetry together with the absence of space inversion supports forbidden transitions. Formally, the *d-d* electro-dipole transitions are symmetry allowed between ground state levels in each Co^{2+} site and all other excited split levels. The spin-orbit coupling between states of $3d^7$ configuration and states of other configurations provides the mechanism for its non-zero intensity. The most intensive *d-d* bands are expected between levels belonging to the initial terms of the free ion with the same multiplicities, so called "spin allowed *d-d* transitions". Due to their intensity, these lines can be considered characteristic of a given ion and its crystal field environment. In the case of Co^{2+}, these transitions can occur between internal levels belonging to the ^4F term (the ground state term of the free ion), as well as between the ^4F and ^4P levels. In case of highly anisotropic g-factors of the ground state Kramers doublet, the *d-d* transitions may occur between terms of different multiplicity, so-called "spin-forbidden *d-d* transitions", with the only requirement $\Delta S_z=0$. In both the allowed and forbidden cases, the intensity of any transition depends on the relative contributions to the wave function, from wave functions of the other configurations as determined by the spin-orbit coupling.

Note, that under low symmetry conditions, the Z_{eff} value is formed by the real position of the ligands and therefore includes asymmetric terms of conventional crystal field theory. The use of a complete set of orthonormal multi-determinant wave functions of the $3d^n$ shell, including spin-orbit interaction, ensures the high accuracy of the MCFT calculations. The advantage of the MCFT theory is that it considers the renormalization of the spin-orbit coupling compared to the value for a free ion for the same reason that the effective nuclear charge is renormalized. The spectra demonstrate a full splitting that is driven by the low site symmetry.

Under MCFT calculations, we used the crystallographic data from Ref. [S3] for positions of the metal ions and the ligands (with valence state 2e) for the β-phase $CoMoO_4$ powder and data for lattice constants of our β-phase thin film at 12 K from Table S1. The energy diagrams show small but visible differences in the crystal field environments (i.e. in metal and ligand positions) between both coordination complexes; this is for instance seen as a difference in the value of splitting of levels originating from ^4F and ^4P terms shown in red colour in Figure 7 of the main manuscript. Comparing the calculated results with the experimentally observed *d-d* band transitions at 0.86, 2.11, and 2.65 eV in the β-phase of the $CoMoO_4$ film demonstrate that they are most accurately aligned with the experimental ones at $Z_{eff} = 6.925|e|$, for Co^{2+} in *4h* positions and $Z_{eff} = 6.975|e|$, for Co^{2+} in *4i* positions.

The theoretical assignment of all lines at respective Z_{eff} is presented in Table S2. The primary assignment is based on the approximately conserved spin multiplicity S for almost all lines. The azimuthal momentum *L* and total angular momentum *J* are poor quantum numbers in this case, due to the low symmetry of the crystal field and spin-orbit contribution. For the same reason, the subsequent assignment to purely octahedral strongly degenerate irreducible representations is very approximate due to the presence of completely split levels. The numbers in bold black colour in Table S2 denote energies of the experimentally observed *d-d* transitions.

We have made similar calculations of the Co^{2+} energy diagrams for the β-phase of a powder sample of $CoMoO_4$ with ion positions and lattice constants described in Ref. [S3] (not shown). We found that the best fit of the observed *d-d* spectra at 0.84, 1.76, and 2.13 eV occurs with $Z_{eff} = 7|e|$ for both Co^{2+} sites (see Table 1 in the manuscript). These values are in accordance with expected lower crystal field strength in a powder sample compared to our strained film.

Interestingly, restoring of the Z_{eff} in the normal α-phase of the $CoMoO_4$ using the experimental characteristic line at 0.88 eV and crystallography data (Ref. [S3]) illustrates that the crystal field strength is almost the same for the Co(1) in the α-phase of $CoMoO_4$ as for the β-phase of the strained $CoMoO_4$ film, but stronger for Co(2) in the normal α-phase compared to the β-phase of the strained film. Its expected modification in the normal α-phase unit cell volume remains lower than in the β-phase of the strained film.

Finally, we illustrate how "spin-forbidden *d-d* transitions" involving transitions between d-levels with different multiplicity can be realized in the case of highly asymmetric crystal field environments. For example, the g-factor for the splitting of the Co(1) first Kramers doublet in a magnetic field written along the main axes has the form:

$$G(12) = \begin{pmatrix} 7.0793 & 0 & 0 \\ 0 & 2.1805 & 0 \\ 0 & 0 & 0.62685 \end{pmatrix}$$

This result is evidence that at a particular polarization of the incoming irradiation one can achieve the necessary condition $\Delta S_z=0$ for *d-d* transitions between the ground state Kramers doublet originated from the term with S=3/2 and the Kramers doublets originated from terms with S=1/2.

Rough octahedral assignment	Co^{2+}(1), Z_{eff}=6.925 (12K)			
	Parameters and energy of 38 lowest Kramers doublets			
	< L >	< S >	< J >	E(eV)
$^4T_{1g}$(F)	2.9	1.5	3.8	0
	2.9	1.5	3.6	0.0088
	3	1.5	4	0.1226
	3	1.5	3.5	0.1492
	3	1.5	3.4	0.2012
	3	1.5	3	0.2234
$^4T_{2g}$(F)	**3**	**1.5**	**3.4**	**0.8628**
	**3**	**1.5**	**3.4**	**0.8665**
	3	1.5	3.6	1.0422
	3	1.5	3.5	1.0479
	3	1.5	3.5	1.1246
	3	1.5	3.3	1.1344
^{2}G	3.9	0.6	4	1.1412
	3.9	0.5	4.1	1.2345
$^4A_{2g}$(F)	2.7	1.5	3.1	1.8526
	2.7	1.5	3.1	1.8551
^{2}G	4.4	0.6	4.6	1.8728
	4	0.5	4.2	2.0107
	3.9	0.5	4	2.0448
	4.1	0.5	4.3	2.066
	**4**	**0.5**	**4.1**	**2.1064**
	**4.1**	**0.5**	**4.1**	**2.1359**
$^4T_{1g}$(P)	1.6	1.4	2.3	2.3559
	1.5	1.5	2.3	2.3706
^{2}F	3	0.6	3.2	2.5363
	2.8	0.7	3	2.5479
	3	0.6	3.1	2.6186
$^4T_{1g}$(P)	**1.8**	**1.5**	**2.5**	**2.7505**
	**1.8**	**1.4**	**2.5**	**2.7582**
^{2}G	3.9	0.5	4	2.7825
	4.5	0.6	4.6	2.9326
^{2}F	3.3	1	3.6	2.9587
$^4T_{1g}$(P)	2.6	1.2	2.9	2.9732
	2.4	1.2	2.8	2.9911
^{2}G	4.1	0.7	4.2	3.0699
	4.3	0.5	4.4	3.1313
	4.1	0.5	4.2	3.3079
	3.9	0.5	4	3.3299

Co^{2+}(2), Z_{eff}=6.975 (12K)				Rough octahedral assignment
Parameters and energy of 38 lowest Kramers doublets				
< L >	< S >	< J >	E(eV)	
2.9	1.5	3.7	0	$^4T_{1g}$(F)
2.9	1.5	3.5	0.0113	
3	1.5	3.8	0.2235	
3	1.5	3.4	0.2378	
3	1.5	3.5	0.3338	
3	1.5	3.3	0.3431	
**2.9**	**1.5**	**3.3**	**0.8623**	$^4T_{2g}$(F)
**2.9**	**1.5**	**3.3**	**0.8641**	
3.9	0.5	4.1	0.9646	^{2}G
3	1.5	3.5	1.2223	$^4T_{2g}$(F)
3	1.5	3.5	1.2245	
3	1.5	3.5	1.3198	
3	1.5	3.4	1.3298	
4	0.5	4.1	1.5055	^{2}G
4.1	0.5	4.3	1.835	
4.2	0.5	4.3	1.9004	
2.4	1.5	2.9	1.9463	$^4A_{2g}$(F)
2.4	1.5	2.9	1.9521	
4.2	0.5	4.3	2.0333	^{2}G
3.6	0.5	3.7	2.2077	
3.9	0.5	3.9	2.2542	
4.3	0.5	4.4	2.3388	
3.5	0.6	3.6	2.4125	
2.4	1.2	2.7	2.528	$^4T_{1g}$(P)
2	1.5	2.7	2.5601	
2.2	1.1	2.8	2.5958	
**1.9**	**1.3**	**2.4**	**2.6608**	
2	1.3	2.4	2.7076	
3.1	0.8	3.4	2.7675	^{2}G
4.2	0.5	4.3	2.9029	
4.2	0.5	4.2	2.9104	
4	0.5	4.1	3.2394	
4.4	0.5	4.5	3.2648	
3.9	0.6	4	3.3773	
3.7	0.7	3.8	3.3915	
2.7	1.2	3.1	3.4373	
1.6	1.4	2.3	3.4542	$^4T_{1g}$(P)
3.6	0.8	3.8	3.5314	^{2}G

Table S2. Theoretical assignment of all spectral lines at the respective Z_{eff} values of Co(1) and Co(2) of β-$CoMoO_4$ thin films. The numbers in bold black colour represent the energies of the experimentally observed *d-d* transitions.

References

S1. J. Williamson, G. V. M. Williams and S. V. Chong "Spin flop transitions and complex magnetic behaviour in $CoMoO_4$ powders and β-$CoMoO_4$ thin films," Journal of Magnetism and Magnetic Materials, vol. 603, p. 172214 (2024)

S2. V. Ksenofontov, Yu. G. Pashkevich, M. Panthöfer, V. Gnezdilov, R. Babkin, R. Klauer, P. Lemmens, and A. Möller "$FeMoO_4$ Revisited: Crosslike 90° Noncollinear Antiferromagnetic Structure Caused by Dzyaloshinskii–Moriya Interaction," The Journal of Physical Chemistry C, vol. 125, p. 5947–5956 (2021)

S3. L. C. Robertson, M. Gaudon, S. Jobic, P. Deniard and A. Demourgues "Investigation of the First-Order Phase Transition in the $Co_{1-x}Mg_xMoO_4$ Solid Solution and Discussion of the Associated Thermochromic Behavior," Inorganic Chemistry, vol. 50, p. 2878-2884 (2011)
